# Categorical Representation of Numerosity in the Pigeon Entopallium: Coding Format and Temporal Dynamics

**DOI:** 10.3390/ani16162494

**Published:** 2026-08-11

**Authors:** Peng Wu, Yanyan Peng, Juncai Zhu, Qingzhi He, Jiangtao Wang, Xiaoke Niu, Songwei Wang, Zhizhong Wang, Li Shi

**Affiliations:** 1 School of Electrical and Information Engineering, Zhengzhou University, Zhengzhou 450001, China; pengwu@gs.zzu.edu.cn (P.W.); yypeng@gs.zzu.edu.cn (Y.P.); zhujuncai@gs.zzu.edu.cn (J.Z.); hqz@gs.zzu.edu.cn (Q.H.); jiangtaowang@zzu.edu.cn (J.W.); niuxiaoke@zzu.edu.cn (X.N.); wangsongwei@zzu.edu.cn (S.W.); 2 Henan Key Laboratory of Brain Science and Brain-Computer Interface Technology, Zhengzhou University, Zhengzhou 450001, China; 3 Department of Automation, Tsinghua University, Beijing 100084, China

**Keywords:** numerosity, single-unit recording, pigeon, entopallium, neural coding, temporal dynamics

## Abstract

Numerosity perception is an evolutionarily conserved ability observed across diverse taxa, from insects and fish to birds and primates. Unlike traditional visual categories, numerosity possesses an intrinsic metric structure, making it well suited for quantitatively investigating how categorical representations emerge along the visual hierarchy. While numerical representations are well characterized in high-level associative areas of non-human primates and avian species, neuronal processing of numerosity in upstream regions remains largely unexplored. To address this gap, we recorded single-unit activity in the pigeon entopallium during a delayed match-to-numerosity task. We found a subset of neurons that encoded numerosity independently of the non-numerical feature controlled in the corresponding recording session, exhibiting quasi-monotonic increasing (QMI) or decreasing (QMD) response profiles. Compared with QMI neurons, QMD neurons exhibited a markedly later coding window and stronger category discriminability. Moreover, in both neuronal populations, the normalized response functions and category discriminability were better described by compressed numerosity scales than by a linear scale, with the logarithmic scale showing a modest overall advantage. These findings help refine current theoretical frameworks for how numerosity information is extracted and represented along the avian visual processing hierarchy, providing a comparative basis for cross-species research.

## Simple Summary

Across the animal kingdom, the ability to extract numerical information supports ecologically important behaviors, including foraging, predator avoidance, and social decision-making. Pigeons exhibit numerical competence comparable to that of non-human primates, making them well suited for studying this fundamental capacity. To understand how the brain extracts numerical information from visual input, we recorded single-neuron activity in the entopallium, a key visual processing region in the avian brain, while pigeons performed a delayed match-to-numerosity task. We found a subset of neurons that encoded numerosity independently of the non-numerical feature controlled in the corresponding recording session and exhibited either quasi-monotonic increasing or quasi-monotonic decreasing response profiles. Compared with neurons with increasing response profiles, neurons with decreasing response profiles showed later numerosity-related activity and stronger numerical-category discrimination. Furthermore, the encoding properties of both neuronal populations were better described by compressed rather than linear numerosity scaling. Together, these findings provide neuronal-level evidence that helps us understand the neural basis of numerical competence in birds.

## 1. Introduction

Numerosity, the number of elements in a set, is a fundamental aspect of environmental perception observed across diverse taxa [1,2,3,4,5,6]. The ability to extract numerical information supports a wide range of ecologically relevant behaviors, such as foraging [7,8], predator avoidance [9,10], and social decision-making [11,12]. Beyond its ecological significance, numerosity possesses an intrinsic metric structure that renders differences between category levels quantifiable, providing a powerful framework for quantitatively investigating categorical processing along the visual hierarchy. In non-human primates, research on the neuronal representation of numerosity has focused primarily on the prefrontal cortex (PFC) and the posterior parietal cortex (PPC) [13,14,15]; in birds, such research has centered on the nidopallium caudolaterale (NCL) [16,17,18,19,20,21], widely regarded as the functional analogue of the PFC [22,23]. Across these high-level associative areas, number neurons exhibit remarkably similar response properties: they respond maximally to their preferred numerosity, with firing rates decreasing progressively as numerical distance from the preferred value increases, consistent with a labeled-line code [18,21,24]. Moreover, their tuning functions are best described on a logarithmic numerosity scale, consistent with the Weber–Fechner law [15,16,25]. While studies in these high-level associative areas have provided a detailed characterization of downstream stages of numerical processing, the neuronal processing of numerosity in regions upstream of these associative areas remains largely unexplored. This knowledge gap limits our understanding of how numerosity information is extracted and represented along the visual processing hierarchy.

Pigeons exhibit remarkable numerical competence [26], making them well suited for investigating the categorical representation of numerosity. To address the gap regarding the upstream extraction process, we targeted the entopallium (ENTO), a major thalamorecipient zone of the avian tectofugal visual system [27,28]. In contrast to mammalian early visual cortex (e.g., V1–V3), ENTO neurons show little or no orderly retinotopic organization and typically have large receptive fields that span broad regions of the visual field [29,30]. The ENTO is located in the dorsal ventricular ridge (DVR) of the telencephalon and sends projections to the dorsally adjacent nidopallium intermedium (NI) and the mesopallium ventrolaterale (MVL), both of which in turn provide input to the NCL [31,32,33]. In zebra finches, anatomical tracing studies have also revealed direct projections from ENTO to NCL [34].

**Figure 1:**
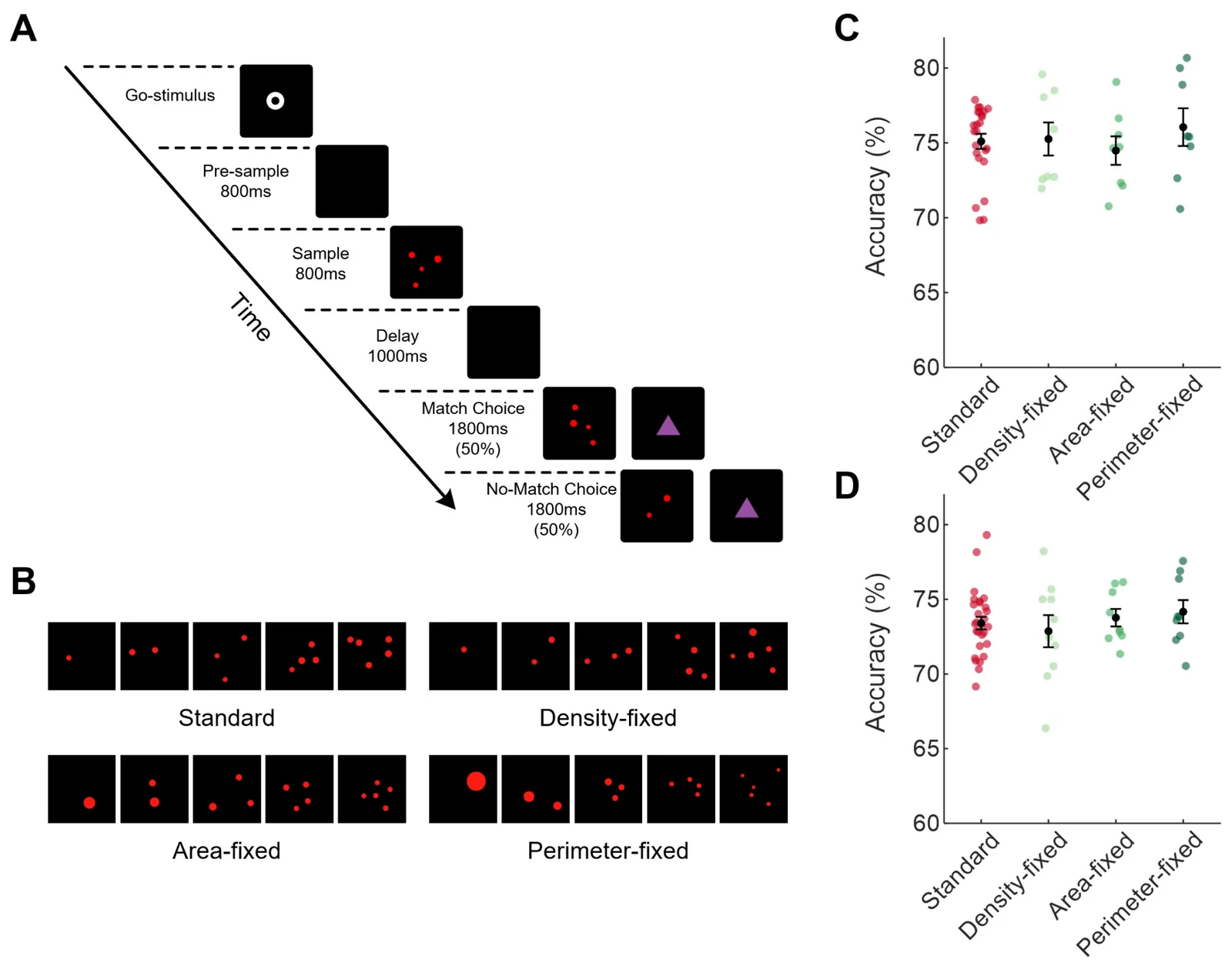
Task design, numerosity stimuli, and behavioral performance. (**A**) Delayed match-to-numerosity task. During the choice period, pigeons chose between a test stimulus and a purple triangle. Selecting the test stimulus was rewarded when its numerosity matched the sample, whereas selecting the purple triangle was rewarded when the two numerosities differed. (**B**) Representative examples of the standard stimuli and the three types of control stimuli. (**C**,**D**) Behavioral accuracy of Pigeon A and Pigeon B during neural recording sessions for the standard and three control stimulus sets. Points represent individual recording sessions; black markers and error bars indicate mean ± SEM.

To characterize neuronal responses to numerosity, we used a delayed match-to-numerosity task adapted from the paradigm established in our previous behavioral study [35] (Figure 1A). Each experimental session included one standard stimulus set and one control stimulus set to reduce the likelihood that pigeons relied on low-level non-numerical visual features to solve the task [16,36]. Across sessions, three types of control stimuli were used: “Density-fixed,” “Area-fixed,” and “Perimeter-fixed” (Figure 1B). After achieving stable behavioral performance, we performed single-unit recordings in the ENTO to examine neuronal responses to numerosity stimuli, thereby providing empirical evidence to refine current theoretical frameworks for how numerosity information is extracted and represented along the avian visual processing hierarchy.

## 2. Materials and Methods

### 2.1. Subjects

Two adult pigeons (*Columba livia*) were obtained from a colony maintained in our laboratory animal facility. Before formal training on the delayed match-to-numerosity task, both pigeons had undergone only delayed matching-to-sample pretraining and had not participated in any other behavioral tasks. Behavioral training was conducted using the fully automated apparatus described in our previous behavioral study [35]. Food rewards were delivered only following correct choices. Each session was terminated after 10 consecutive min without completion of a trial, which was taken to indicate presumed satiation and loss of task motivation. Water was available ad libitum, and the animals were maintained under a 14:10 h light/dark cycle. Supplemental grit (1.2 g) was provided after each behavioral session as part of routine animal care. Before electrode implantation surgery, the pigeons were transferred to an operant conditioning chamber for habituation to the recording environment.

### 2.2. Behavioral Task and Numerosity Stimuli

Each recording session comprised a continuous period of task performance with concurrent electrophysiological recording. During each session, the pigeons performed a delayed match-to-numerosity task adapted from the previously described behavioral paradigm [35] (Figure 1A). Behavioral choices were detected using a customized infrared sensing frame positioned 2 cm in front of the screen surface, allowing response detection without physical contact between the pigeon’s beak and the screen. Each trial began when the pigeon pecked a central circular cue. After an 800-ms pre-sample period, a sample stimulus was presented for 800 ms, followed by a 1000-ms delay period. If a peck was detected during the sample or delay period, the trial was immediately terminated, and a correction trial was presented after a 5–8 s time-out. During the choice period, a test stimulus and a purple triangle were presented simultaneously. On match trials (50%), the test stimulus contained the same numerosity as the sample, and pigeons were rewarded for selecting the test stimulus. On non-match trials (50%), the test stimulus differed in numerosity from the sample, and pigeons were rewarded for selecting the purple triangle. A response window of 1800 ms was imposed for the choice period. Incorrect choices or response omissions were followed by correction trials after the 5–8 s time-out. The reported number of trials per session included only initial trials completed with a choice response, and behavioral accuracy was calculated as the proportion of correct responses among these trials. Incomplete initial trials and all correction trials were excluded from trial counts, accuracy calculations, and single-unit analyses.

Numerosity stimulus images (Figure 1B) were generated using a custom script written in MATLAB R2022a (MathWorks, Natick, MA, USA). The diameter of individual dots on the screen ranged from 1 to 7 mm. Each recording session included one standard stimulus set and one of three control stimulus sets (“Density-fixed,” “Area-fixed,” or “Perimeter-fixed”). To obtain sufficient trials for each numerosity × stimulus-type condition, a single control set was used per session, with the control type alternated across sessions [16]. These control sets held average density, total area, or total perimeter constant, respectively, to reduce the likelihood that pigeons relied on low-level non-numerical visual features to solve the task [16,37]. To minimize the possibility that pigeons solved the task by memorizing specific visual configurations, we generated a new set of 60 numerosity stimulus images for each session, including 30 standard and 30 control images. Although any image could serve as either a sample or a test stimulus, the sample and test displays within a single trial were never physically identical, including in numerosity-matching trials. Trial parameters, including numerosity, trial type (match vs. non-match), and stimulus type (standard vs. control), were pseudo-randomized across trials.

### 2.3. Surgery and Recordings

Electrode implantation surgery was performed under general anesthesia with sodium pentobarbital (1.5% solution, 2 mL/kg) and perioperative analgesia with butorphanol (1 mg/kg). Feathers over the head and around the ears were trimmed, and the pigeon was secured in a stereotaxic apparatus (RWD Life Science Co., Ltd., Shenzhen, China) aligned with the coordinate system defined in the Karten and Hodos pigeon brain atlas [38]. Topical 2% lidocaine was applied to the scalp for local anesthesia. The scalp was then incised to expose the skull, and a craniotomy was opened, centered at anteroposterior (AP) 10.0 mm and mediolateral (ML) 6.0 mm left of the midline. A custom adjustable microdrive electrode array (4 × 4 tungsten microelectrodes, 300 μm spacing; 2-mm travel range; KD-MEA-AD; Ketou Brain-Machine Technology Co., Ltd., Suzhou, China) was implanted through the craniotomy. All implantation procedures were performed under a surgical microscope (LZJ-4D; ZTGX Optical Instrument Co., Ltd., Zhenjiang, China).

Before each recording session, the microelectrode array was further advanced to a new recording depth until well-isolated single-unit activity was detected on at least one channel; neurons were not preselected based on task relevance. Neural signals were acquired using the Cerebus™ Neural Processing System (Blackrock Microsystems, Inc., Salt Lake City, UT, USA). A headstage containing a preamplifier was plugged into the connector of the microelectrode array, and the signals were further amplified (gain, 300×) using a multichannel amplifier. Task-event markers were generated by the main control module of the fully automated training apparatus. The markers were transmitted via a serial interface (PL2303HXD; Xinwei Electronic Technology Co., Ltd., Shenzhen, China) to an analog input channel of the Cerebus™ data acquisition system and were recorded synchronously with the neural signals. This synchronization enabled precise alignment of neuronal activity to the corresponding task events.

Spike sorting was performed offline using Wave_Clus 3 [39,40]. Initial clusters were generated based on spike waveform features and were subsequently inspected manually. Waveform signal-to-noise ratio (SNR) was calculated as $SNR=A_{peak}/SD_{noise}$, where $A_{peak}$ is the absolute peak amplitude of the mean spike waveform, and $SD_{noise}$ is the standard deviation of the band-pass-filtered data trace (300–5000 Hz). Clusters were classified as putative single units and included in subsequent analyses only if they showed stable waveform morphology, clear separation from noise and neighboring clusters, and a waveform SNR of at least 3:1. Clusters that did not meet these isolation criteria were excluded from further analyses. For each included unit, the refractory-period violation rate was defined as the proportion of inter-spike intervals (ISIs) shorter than 1.5 ms across the entire recording period. Across the included putative single units, this rate was 0.47 ± 0.54% (mean ± SD).

### 2.4. Histology

After completion of the recording sessions, the pigeons were euthanized with an overdose of sodium pentobarbital and transcardially perfused with phosphate-buffered saline (PBS) followed by 4% paraformaldehyde (PFA). The brains were then removed, post-fixed in 4% PFA for 12 h, and cryoprotected in 30% sucrose solution at 4 °C for 48 h. Subsequently, frozen coronal sections (30 μm) were cut using a cryostat and mounted on gelatinized slides. The sections were Nissl-stained and mapped onto the standard brain atlas of Karten and Hodos [38] to verify the recording sites (Supplementary Figure S1).

Functional heterogeneity in visual processing has been reported within the pigeon entopallial complex [41]. Previous anatomical studies have also used different criteria and nomenclatures to subdivide the pigeon entopallial complex. In the classical ectostriatal terminology, this region was divided into the central core of the ectostriatum (Ec) and the peri-ectostriatal belt (Ep), with some connectional studies further identifying an adjacent Ep2 region immediately surrounding Ep [31,42,43]. In another anatomical framework based on histological characteristics and differential projection patterns, the entopallium proper is subdivided into the entopallium internum (Ei) and entopallium externum (Ex), whereas the perientopallium (Ep) forms a relatively narrow belt surrounding the entopallium proper [27]. The subdivision boundaries defined under these anatomical frameworks are not fully equivalent, and routine Nissl-stained sections alone cannot reliably delineate the boundaries of these subdivisions. More precise subdivision-level localization would require additional anatomical markers, such as cytochrome oxidase (CO) histochemistry and parvalbumin (PV) immunohistochemistry. Moreover, the electrode array was manually advanced across sessions until well-isolated single-unit activity was detected, and the advancement depth was not fixed. We therefore did not assign individual recorded units to specific entopallial subdivisions.

### 2.5. Data Analysis

For single-unit analyses, units were included only if they had ≥10 correct trials in each numerosity × stimulus-type condition (mean, 46 correct trials per condition) and an overall mean firing rate ≥ 1 Hz across the trial. In the present study, the sample stimulus was presented from 0 to 800 ms. To account for visual response latency, and because the precise visual response latency of pigeon ENTO has not been established, we adopted an estimated 50–100 ms latency correction based on previous electrophysiological studies of the avian tectofugal pathway and related visual forebrain regions [16,44,45]. Accordingly, the sample-related analysis period was defined as 50–900 ms relative to sample onset.

Neuronal firing rates were then analyzed using a two-factor sliding-window ANOVA, with numerosity (five levels: 1–5) and stimulus type (two levels: standard and control) treated as categorical factors (200-ms window, 10-ms step; significance threshold, *p* < 0.01). To distinguish numerosity-related activity from general visually evoked responses, neurons were classified as numerosity selective only when their firing rates varied systematically with numerosity and this effect generalized across the standard stimulus set and the session-specific control stimulus set. Specifically, a neuron was classified as numerosity selective if, within the sample-related analysis period, it showed a significant main effect of numerosity for ≥11 consecutive windows (corresponding to a continuous interval of at least 300 ms), with no significant main effect of stimulus type and no significant numerosity × stimulus-type interaction over the same windows. Units showing a significant stimulus-type effect, a significant numerosity × stimulus-type interaction, or lacking a sustained numerosity effect that met the consecutive-window criterion were not classified as numerosity-selective neurons and were excluded from subsequent analyses of numerosity-selective activity. For each neuron classified as numerosity selective, numerosity-selectivity latency was defined as the onset of the earliest interval satisfying the above criteria. If multiple such intervals were present, the longest was retained as the selectivity window for subsequent response analyses.

To account for temporal dependence among overlapping windows, the retained selectivity interval was additionally evaluated using a maximum-cluster-length permutation test. For each of 1000 permutations, trial-wise numerosity labels were shuffled within stimulus type, the same shuffled labels were applied across all time windows, and the two-factor sliding-window ANOVA was recomputed. The maximum number of consecutive windows meeting the predefined numerosity-selectivity criteria was recorded for each permutation to generate the null distribution. The cluster-level permutation *p* value was calculated as $p_{perm}=(N_{perm\;\geq \;obs}+1)/(N_{perm}+1)$, where $N_{perm\;\geq \;obs}$ is the number of permutations in which the maximum cluster was at least as long as the observed interval, and $N_{perm}$ is the total number of permutations (1000). A cluster-level permutation *p* value < 0.05 was considered significant. Based on the overall response trend within the selected window, numerosity-selective neurons were further classified as quasi-monotonic increasing (QMI) or quasi-monotonic decreasing (QMD). For each QMI or QMD neuron, the mean firing rate for each numerosity was calculated within its selected window and normalized by setting the maximum response to 100% and the minimum response to 0%. The normalized response profiles were then averaged within each group to obtain the population-mean response functions for the QMI and QMD populations.

We quantified the linearity of the normalized response profiles across four candidate numerosity scales: a linear scale, two power scales with exponents of 0.5 and 0.33, and a logarithmic scale. These candidate scales span different degrees of numerosity compression and are commonly used to evaluate the putative representational scale of numerical magnitude in high-level associative areas [16,18,46]. In the present analysis, we sought to determine which of these scales best linearized the response profiles of QMI and QMD neurons. For each candidate numerosity scale, an ordinary least-squares (OLS) linear regression model was fitted to the normalized response profile of each neuron. Goodness of fit was quantified using the coefficient of determination (*R*^2^) and root-mean-square error (RMSE).

For the selectivity-window analyses, we quantified each neuron’s discriminability for all numerosity pairs within its selectivity window using the area under the receiver operating characteristic curve (AUROC). AUROC was then transformed into signed AUROC (sAUROC), defined as sAUROC = 2 × (AUROC − 0.5), with values ranging from −1 to 1. For each pair, the larger numerosity was assigned to the positive class. Thus, sAUROC > 0 indicates higher firing rates for the larger numerosity, whereas sAUROC < 0 indicates higher firing rates for the smaller numerosity. Values close to 0 reflect weak discriminability, whereas values approaching −1 or 1 reflect strong discriminability. The five numerosities (1–5) yield 10 unique numerosity pairs. We visualized each neuron’s discriminability across all pairs using a pairwise sAUROC graph, in which node color indicates numerosity, and the color of connecting lines denotes the corresponding sAUROC value. Warm colors indicate sAUROC > 0, cool colors indicate sAUROC < 0, and greater color saturation reflects larger absolute sAUROC values.

To enable direct comparisons of discriminability between QMI and QMD neurons, we defined a direction-aligned sAUROC (DA-sAUROC): DA-sAUROC = sAUROC for QMI neurons and −sAUROC for QMD neurons. Subsequently, for each numerosity pair $\left(n_{1},n_{2}\right)$ with $n_{1},n_{2}\in \left\{1,\;2,\;3,\;4,\;5\right\}$ and $n_{1}\neq n_{2}$, we defined the per-unit-distance discriminability on a candidate numerosity scale s as:

(1) $${UD}_{s}\left(n_{1},n_{2}\right)=\frac{\mathrm{DA}\text{-}\mathrm{sAUROC}\left(n_{1},n_{2}\right)}{d_{s}\left(n_{1},n_{2}\right)}$$

where $d_{s}\left(n_{1},n_{2}\right)$ denotes the numerical distance between $n_{1}$ and $n_{2}$ on scale s:

(2) $$d_{s}\left(n_{1},n_{2}\right)=\left|g_{s}\left(n_{1}\right)-g_{s}\left(n_{2}\right)\right|$$

Here, $g_{s}\left(\cdot \right)$ denotes the transformation function for the candidate scale s. For each QMI and QMD neuron, we computed ${UD}_{s}\left(n_{1},n_{2}\right)$ for all 10 numerosity pairs on each candidate scale, and then averaged the results within the respective populations to obtain the population-mean ${UD}_{s}\left(n_{1},n_{2}\right)$.

To quantify the uniformity of per-unit-distance discriminability across numerosity pairs, we calculated the coefficient of variation (CV) of the population-mean $UD_{s}(n_{1},n_{2})$ values across the 10 numerosity pairs for each candidate scale and neuronal population. Lower CV values indicate greater uniformity of per-unit-distance discriminability across numerosity pairs.

Together, numerosity-axis compression in QMI and QMD neuronal activity was assessed using two complementary analyses. First, in the response-profile analysis, we examined which candidate numerosity scale best linearized the normalized response profiles. Second, in the discriminability analysis, we examined which candidate scale produced the most uniform distribution of per-unit-distance discriminability across numerosity pairs.

To characterize the temporal evolution of numerosity discriminability across the entire numerosity-selective population, we additionally computed sAUROC for each of the 10 numerosity pairs in each numerosity-selective neuron using a 200-ms sliding window advanced in 10-ms steps and direction-aligned the resulting values as defined above.

To quantify the dynamic encoding of numerosity, stimulus protocol, and their interaction in the QMI and QMD populations during the sample-related analysis period, we computed the percent-explained variance (PEV; *ω*^2^) using a sliding-window approach (200-ms window, 10-ms step). Within each window, a two-way ANOVA was applied to the firing rates of individual neurons to extract *ω*^2^ for each main effect and their interaction. Finally, these single-neuron *ω*^2^ values were averaged across the respective populations, yielding population-level PEV time courses for each effect.

Following Paul et al. [47], we computed first-harmonic aggregate Fourier power for the numerosity stimuli to test whether ENTO responses were better explained by numerosity or by this measure, which has previously been associated with responses in human early visual cortex (V1–V3). For each image, aggregate Fourier power was calculated by summing the Fourier power spectral density across spatial frequencies within the first harmonic. Because the first-harmonic boundary can be ambiguous when dot sizes vary substantially within an image, this measure was calculated only for images showing a clearly identifiable local minimum in the power spectral density profile (Supplementary Figure S2). For each numerosity-selective neuron, we fitted two separate linear regression models to the neural responses within its selectivity window, using either log(numerosity) or log(first-harmonic aggregate Fourier power) as the predictor. Goodness of fit was quantified using the coefficient of determination (*R*^2^), allowing direct statistical comparison between the two models.

To compare the extent of error-related modulation between QMI and QMD neurons, we analyzed completed initial trials in which the pigeons made an incorrect choice. For each neuron, the selectivity window defined from correct trials was also used to analyze error-trial responses. Mean firing rates within this window were calculated separately for correct and error trials at each of the five sample numerosities. The correct-trial response profile was normalized using its minimum and maximum responses across the five numerosities, and the same normalization parameters were applied to the error-trial response profile. Overall error-related modulation was quantified for each neuron as the mean absolute difference between the normalized correct- and error-trial responses across the five numerosities. These values were compared between QMI and QMD neurons using a two-sided Mann–Whitney U test.

### 2.6. Population Decoding Analysis

To examine how population-level numerosity decoding evolved over time, we performed a time-resolved pseudo-population decoding analysis using a linear multiclass support vector machine (SVM) classifier. All 191 numerosity-selective neurons were included. Because the neurons were recorded in different sessions, pseudo-populations were constructed by combining trial responses across neurons [48]. For each neuron and numerosity, 30 correct trials were randomly sampled without replacement to construct population response vectors. Firing rates were calculated using a 200-ms sliding window advanced in 10-ms steps. At each time window, the SVM was trained using a one-versus-one classification scheme to discriminate among the five numerosities. Performance was evaluated using 10-fold cross-validation: nine folds were used for training, the remaining fold was used for testing, and each fold served once as the test set. The complete trial-sampling and time-resolved decoding procedure was repeated 100 times. Decoding accuracy was defined as the percentage of test trials assigned to the correct numerosity and was averaged across resampling iterations. Chance-level accuracy was 20%.

Statistical significance of time-resolved decoding was assessed using a one-sided maximum-cluster-length permutation test. Within each of the 100 trial-resampling iterations, numerosity labels were independently shuffled 10 times, while the same shuffled labels were retained across all time windows within each permutation, yielding 1000 shuffled-label decoding time courses. At each time point, the 95th percentile of the shuffled-label accuracy distribution was used as the cluster-forming threshold. Consecutive time points at which the observed mean decoding accuracy exceeded this threshold were grouped into candidate clusters. For each shuffled-label time course, the maximum length of clusters exceeding the same thresholds was recorded to construct the cluster-level null distribution. Cluster-level *p* values were calculated using the same add-one permutation correction described above, and observed clusters with *p* < 0.05 were considered significant.

For the confusion-matrix analysis, classifiers were trained using mean neuronal responses within the predefined sample-related analysis interval (50–900 ms), following the same trial-sampling, linear multiclass SVM, and 10-fold cross-validation procedures described above. The analysis was repeated 1000 times. Predictions for the held-out test trials were pooled across folds and resampling iterations, and the resulting confusion matrix was normalized within each true-numerosity category. Each entry therefore represents the percentage of trials of a given true numerosity classified as each predicted numerosity.

## 3. Results

### 3.1. Behavioral Performance

Behavioral performance was assessed across 52 neural recording sessions (Pigeon A, 24 sessions; Pigeon B, 28 sessions). Mean behavioral accuracy across sessions was 75.2 ± 0.4% for Pigeon A and 73.5 ± 0.3% for Pigeon B (mean ± SEM). The mean number of trials per session was 585 for Pigeon A (range, 438–760) and 648 for Pigeon B (range, 454–789). Behavioral accuracy for pigeon A did not differ significantly across the standard stimulus set and the three control stimulus sets (Figure 1C; Kruskal–Wallis test, *p* = 0.774). Pigeon B showed a similar pattern (Figure 1D; Kruskal–Wallis test, *p* = 0.794). These results indicate that the pigeons generalized their performance across the standard and control stimulus sets, suggesting that their behavioral judgments were not driven by covarying low-level visual features. Together, these results provided a reliable behavioral basis for subsequent analyses of single-unit activity.

### 3.2. Single-Neuron Responses to Numerosity

**Figure 2:**
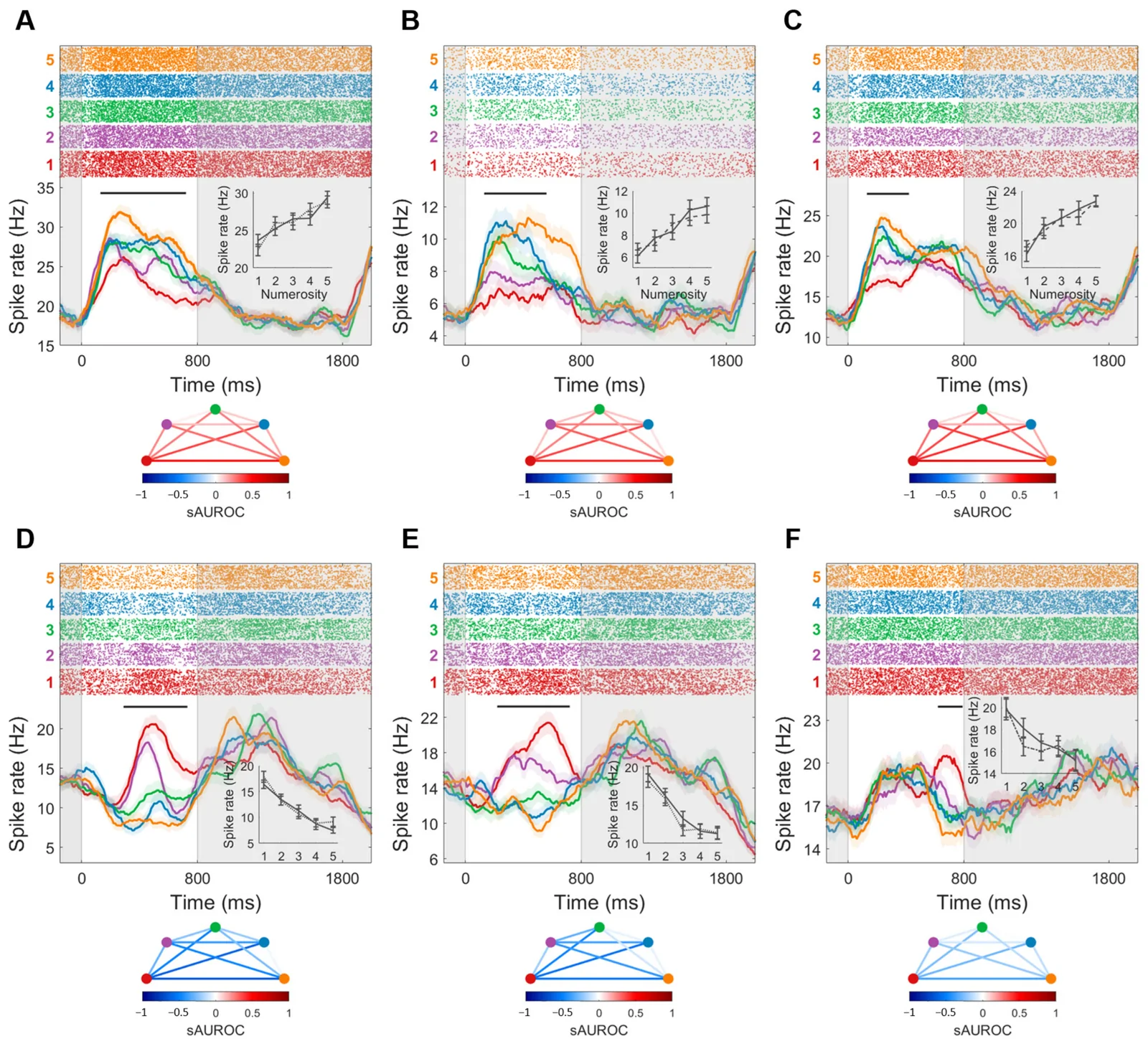
Example responses of QMI (**A**–**C**) and QMD (**D**–**F**) neurons. In each panel: (**Top**) Raster plots showing neural activity. Each row corresponds to one trial, and each dot represents a spike. Colors indicate sample numerosities. (**Middle**) Averaged spike density functions (smoothed with a 100-ms Gaussian kernel). Shaded areas around each curve represent SEM. The transparent window indicates the 800-ms sample stimulus presentation period, and the horizontal black line marks the selectivity time window. (**Bottom**) Pairwise sAUROC graphs illustrating discriminability for each numerosity pair within the selectivity time window. Node color indicates sample numerosity. The color of connecting lines indicates the sAUROC value: warm colors (positive values) indicate that the neuron responds more strongly to the larger numerosity in the pair, whereas cool colors (negative values) indicate stronger responses to the smaller numerosity. Line saturation reflects the strength of discriminability. (Inset) Mean firing rates within the selectivity window for the standard stimuli (solid line) and the session-specific control stimuli. The control set used in each session is denoted by line style: dashed, “Density-fixed”; dotted, “Perimeter-fixed”; and dash-dotted, “Area-fixed.” Error bars represent SEM.

We recorded from 467 single neurons in the ENTO while pigeons performed the numerosity discrimination task. A two-factor sliding-window ANOVA (window-level significance criterion, *p* < 0.01; ≥11 consecutive significant windows) identified 191 neurons (41%) that exhibited a significant main effect of numerosity within specific time windows during the sample-related analysis period, with no significant main effect of stimulus type (standard vs. control) or numerosity × stimulus type interaction in those windows. All 191 neurons also remained significant in a permutation test based on the maximum cluster length (1000 permutations; cluster-level significance criterion, *p* < 0.05). Based on their response trends, these neurons were classified into two groups: quasi-monotonic increasing (QMI) neurons (155/191, 81%) and quasi-monotonic decreasing (QMD) neurons (36/191, 19%). Per-pigeon unit distributions, including QMI and QMD counts, are summarized in Supplementary Table S1. Three example QMI neurons and three example QMD neurons are shown in Figure 2A–C and Figure 2D–F, respectively (see Table 1 for detailed statistics). Within their respective selectivity time windows (horizontal black lines), the example neurons in Figure 2A,B exhibited overall increases in firing rate with numerosity, with similar response profiles for the standard stimuli and the corresponding control stimuli (perimeter-fixed in Figure 2A; density-fixed in Figure 2B). In contrast, the example neuron in Figure 2F exhibited an overall decrease in firing rate with numerosity, with similar response profiles for the standard and area-fixed control stimuli. To further quantify the numerosity discriminability of each neuron, we computed signed AUROC (sAUROC) values for all numerosity pairs within each neuron’s selectivity window (see Section 2), visualized as sAUROC graphs at the bottom of Figure 2A–F.

**Figure 3:**
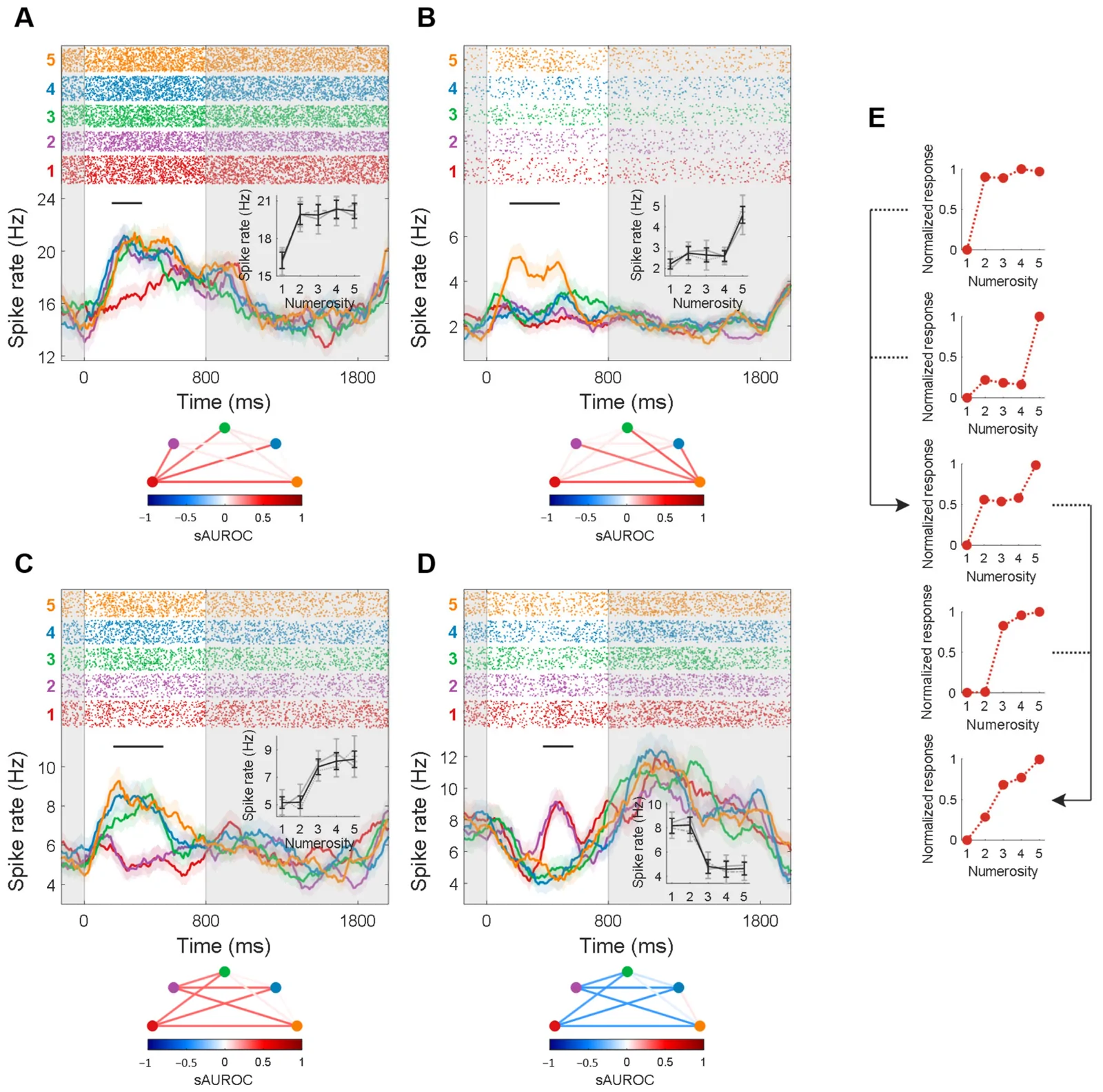
Example responses of coarse category-selective neurons. (**A**) Example neuron showing an overall increasing trend, but with comparable firing rates for numerosities 2–5. The sAUROC graph confirms weak discriminability among these numerosities, suggesting they are grouped into a single category. (**B**) Example neuron exhibiting low, indistinguishable responses to numerosities 1–4, while responding strongly to numerosity 5. (**C**) Example neuron that segments numerosities into two distinct categories (1–2 vs. 3–5). It discriminates well between these two categories but shows little discriminability within each category. (**D**) Example neuron showing a similar categorical boundary to (**C**) (separating 1–2 from 3–5) but with an overall decreasing trend. Insets in (**A**–**D**): The black solid line represents the mean response across the standard and control stimuli within the selectivity time window. Gray solid, dotted, dashed, and dash-dotted lines represent the standard stimuli and the three types of control stimuli, respectively (line styles as in Figure 2). Error bars represent SEM. (**E**) Composite example of coarse category-selective neurons. The first, second, and fourth subpanels show the normalized responses for the neurons in (**A**–**C**), respectively.

**Figure 4:**
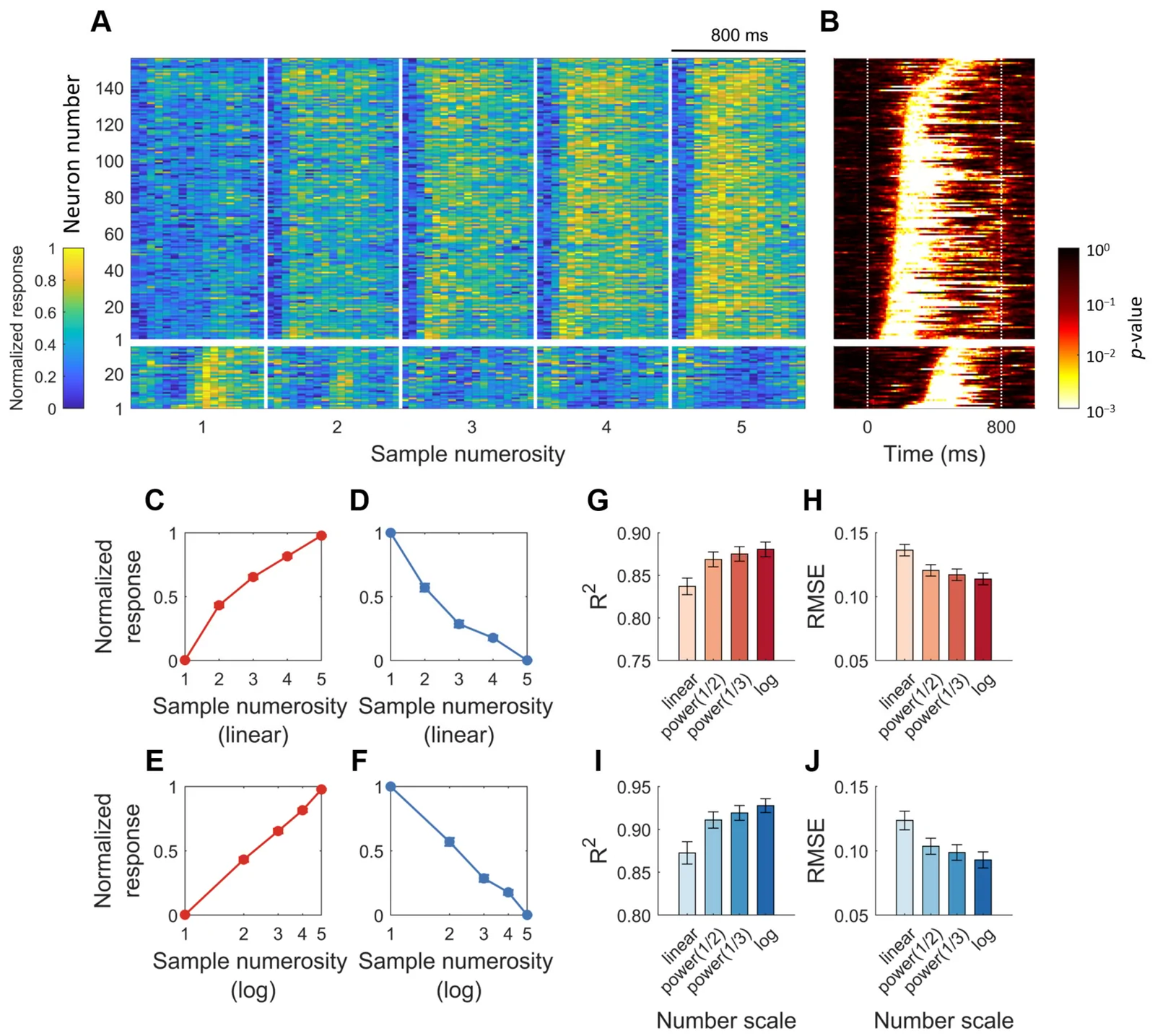
Response properties of QMI and QMD neurons. (**A**) Normalized responses of QMI (**top**) and QMD (**bottom**) neurons to each sample numerosity. Each row represents a single neuron; color represents mean firing rates computed in 50-ms bins during the sample period and normalized to [0, 1] for each neuron. The 50-ms bins were used for visualization only. Sample numerosities are separated by vertical white lines. (**B**) Time-resolved color maps of *p*-values for the numerosity factor, computed using a two-factor sliding-window ANOVA with a 200-ms window and 10-ms step. Neurons in (**A**,**B**) correspond one-to-one and are sorted by the onset of their respective selectivity windows used for response analyses. (**C**,**D**) Mean normalized responses of QMI (**C**) and QMD (**D**) neurons on a linear numerosity scale. (**E**,**F**) Same data as in (**C**,**D**), but on a logarithmic numerosity scale. Note that the response functions exhibit a compressive non-linearity on the linear scale but are approximately linear on the logarithmic scale. (**G**,**H**) Mean coefficient of determination (*R*^2^) and root-mean-square error (RMSE) for QMI neurons across the four numerical scales. (**I**,**J**) Mean *R*^2^ and RMSE for QMD neurons across the four numerical scales. Higher *R*^2^ and lower RMSE values indicate better linear fits. Error bars represent SEM.

We also found a distinct subset of QMI and QMD neurons that showed little or no discriminability between certain adjacent numerosities (example neurons are shown in Figure 3A–D; see Table 1 for corresponding statistics). Within their respective selectivity time windows, the example neurons in Figure 3A,B exhibited overall increases in firing rate with numerosity but little discriminability among numerosities 2–5 (Figure 3A) or 1–4 (Figure 3B). In Figure 3C,D, numerosities 1–2 and 3–5 can be regarded as two separate categories: both example neurons discriminated well between these categories but exhibited little discriminability within each category. Notably, we found that the response profiles of these coarse category-selective neurons can be combined to yield response functions resembling those of the more commonly observed QMI and QMD neurons; a composite example is shown in Figure 3E.

### 3.3. Population Response Characteristics of QMI and QMD Neurons

The responses of all QMI and QMD neurons are summarized in Figure 4A (upper panel: QMI; lower panel: QMD; for statistical results, see Supplementary Tables S4 and S5). Beyond their opposite response trends, QMD neurons exhibited windows of significant numerosity effect that were systematically delayed relative to those of QMI neurons (Figure 4B). Further analysis showed that the numerosity-selectivity latency (mean ± SD) was 193.2 ± 85.3 ms for the QMI neuron population (*n* = 155) and 390.6 ± 72.5 ms for the QMD neuron population (*n* = 36), and these latencies differed significantly between the two neuron populations (Mann–Whitney U test, *p* < 0.001). This QMI–QMD latency difference remained significant when the two pigeons were analyzed separately (Mann–Whitney U tests, both *p* < 0.001; Supplementary Table S1).

To determine whether QMI and QMD neurons differed in error-related response modulation, we compared correct- and error-trial response profiles within each neuron’s selectivity window (Supplementary Figure S3). The mean magnitude of error-related modulation was numerically greater in QMD than in QMI neurons, but the between-population difference did not reach statistical significance (Mann–Whitney U test, *p* = 0.103).

The mean normalized responses of QMI and QMD neurons on linear and logarithmic numerosity scales are shown in Figure 4C–F. In both neuron populations, response differences increased with numerical distance, reflecting a numerical distance effect. On the linear scale, both populations also showed a numerical magnitude effect: for a given numerical distance, response differences tended to become smaller as sample numerosity increased (Figure 4C,D). In contrast, on a logarithmic scale, these differences became nearly constant across numerosities. Accordingly, the population response functions of both QMI and QMD neurons approached linearity (Figure 4E,F), effectively eliminating the numerical magnitude effect.

To quantify the linearity of neuronal response functions on different numerosity scales, we fitted ordinary least-squares (OLS) linear regression models to the normalized response profiles of individual neurons and assessed the goodness of fit using *R*^2^ and RMSE. The resulting metrics for the QMI and QMD neuron populations are summarized in Figure 4G–J. For the QMI neuron population, the mean *R*^2^ values on the linear, power (1/2), power (1/3), and logarithmic numerosity scales were 0.837, 0.869, 0.875, and 0.881, respectively; corresponding RMSE values were 0.136, 0.121, 0.117, and 0.114 (Figure 4G,H). Friedman tests revealed significant differences in both *R*^2^ and RMSE across the four scales (*n* = 155; *R*^2^: *p* < 0.001; RMSE: *p* < 0.001). Post hoc Wilcoxon signed-rank tests (logarithmic scale vs. each of the other three scales) showed that the logarithmic scale yielded the highest *R*^2^ (all *p* < 0.05) and the lowest RMSE (all *p* < 0.01). A similar pattern was observed for the QMD neuron population. The mean *R*^2^ values on the four numerosity scales were 0.873, 0.911, 0.919, and 0.928, respectively; corresponding RMSE values were 0.124, 0.104, 0.099, and 0.093 (Figure 4I,J). Friedman tests also revealed significant differences in both *R*^2^ and RMSE across the four scales (*n* = 36; *R*^2^: *p* < 0.001; RMSE: *p* < 0.001), and post hoc Wilcoxon signed-rank tests showed that the logarithmic scale yielded the highest *R*^2^ (all *p* < 0.05) and the lowest RMSE (all *p* < 0.05).

To assess whether this scale-fitting pattern was consistent across animals, we repeated the comparisons between the logarithmic scale and the other candidate numerosity scales separately for each pigeon (Supplementary Table S2). In both pigeons and in both QMI and QMD populations, the logarithmic scale provided better fits than the linear scale. Additional pairwise comparisons further showed that the power (1/2) and power (1/3) scales also provided significantly better fits than the linear scale (Supplementary Table S3). By contrast, differences between the logarithmic and power-transformed scales were smaller and not consistently significant in the individual-animal analyses, particularly for the strongly compressive power (1/3) scale (Supplementary Table S2). Together, the pooled and per-animal analyses indicate that the response functions of both QMI and QMD populations were better described by compressed numerosity scales than by a linear scale, with the logarithmic scale showing a modest goodness-of-fit advantage in the pooled analysis.

### 3.4. Numerosity Discriminability in QMI and QMD Neuron Populations

**Figure 5:**
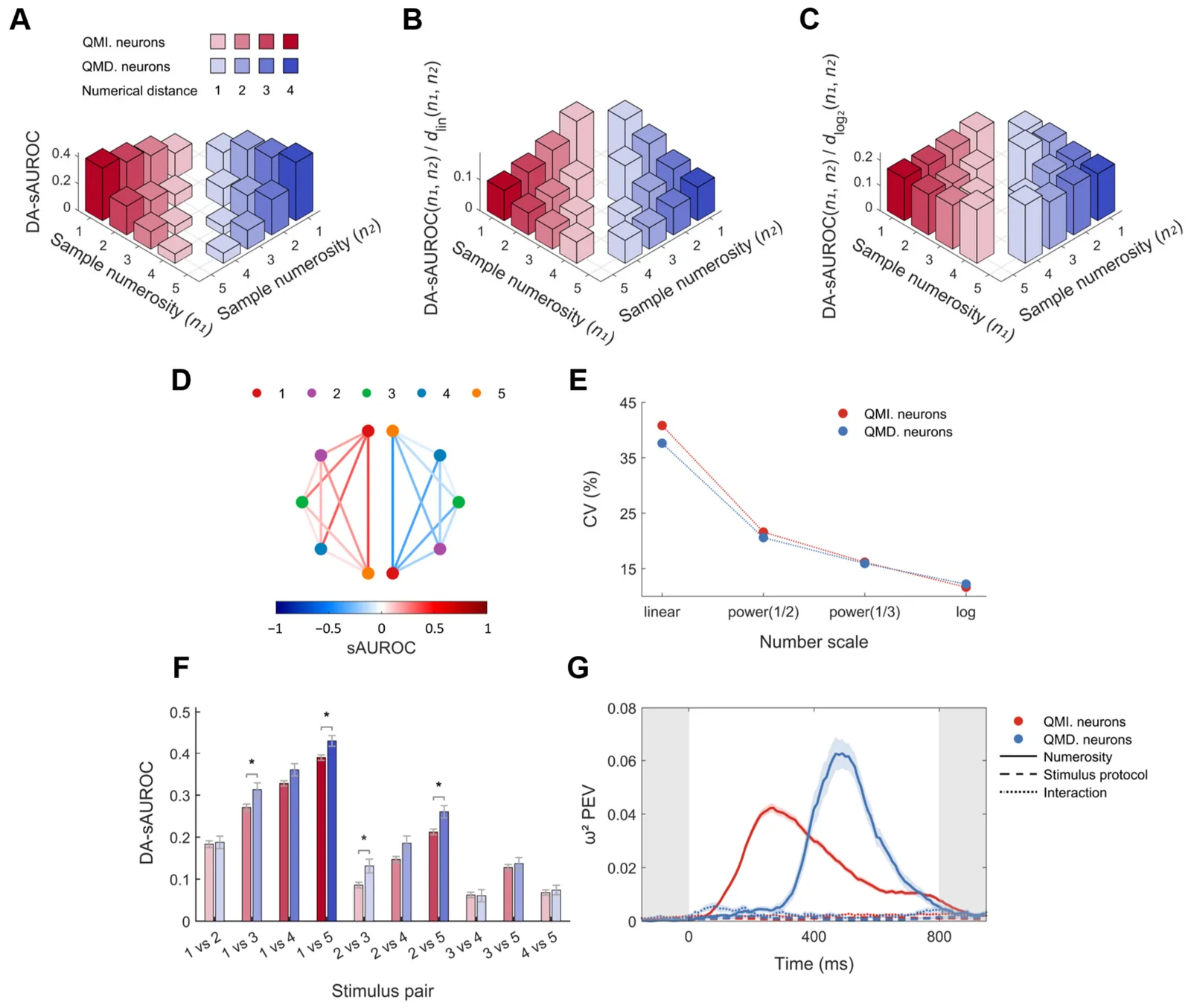
Numerosity discriminability in QMI and QMD neuron populations. (**A**) Population-level discriminability of QMI (left, warm colors) and QMD (right, cool colors) neurons for each numerosity pair. The *x*- and *y*-axes represent the sample numerosities ($n_{1}$ and $n_{2}$), and the *z*-axis displays pairwise discriminability, quantified by DA-sAUROC. (**B**) Population-level per-unit-distance discriminability on the linear scale; $d_{lin}\left(n_{1},n_{2}\right)$ denotes the numerical distance between $n_{1}$ and $n_{2}$ on the linear scale. (**C**) Same as (**B**), but on the logarithmic scale; $d_{{log}_{2}}\left(n_{1},n_{2}\right)$ denotes the numerical distance between $n_{1}$ and $n_{2}$ on the ${log}_{2}$ scale. (**D**) Pairwise sAUROC graphs for QMI (left) and QMD (right) populations. Node color indicates sample numerosity. The color of connecting lines represents sAUROC values, illustrating a clear symmetry of discriminability between the two populations. (**E**) Coefficient of variation (CV) of per-unit-distance discriminability computed across all numerosity pairs for each number scale. (**F**) Comparison of discriminability between QMI and QMD populations for each numerosity pair. Asterisks indicate significant differences (*p* < 0.05, Mann–Whitney U test). Error bars represent SEM. Panels (**A**–**F**) are based on sAUROC values computed within each neuron’s selectivity window. (**G**) Time courses of percent-explained variance (PEV; *ω*^2^) for numerosity, stimulus protocol, and their interaction. Compared with the QMI neuron population, the QMD neuron population showed a markedly later numerosity-coding window and a higher peak PEV for the numerosity factor. Shaded areas around each curve represent SEM.

The mean discriminability for each numerosity pair, computed within each neuron’s selectivity window, is summarized in Figure 5A, revealing approximately symmetric discriminability patterns between the QMI and QMD neuron populations (see also Figure 5D). For each numerosity scale, we computed per-unit-distance discriminability for all numerosity pairs (see Section 2). The results for the linear and logarithmic scales are shown in Figure 5B and 5C, respectively. On the linear scale, per-unit-distance discriminability varied markedly across numerosity pairs in both populations, whereas on the logarithmic scale it remained approximately constant. Further analysis showed that, in both QMI and QMD neuron populations, the coefficient of variation (CV) of per-unit-distance discriminability progressively decreased across the candidate scales, from the linear scale through the power (1/2) and power (1/3) scales to the logarithmic scale (Figure 5E). This pattern indicates that per-unit-distance discriminability was more uniform across numerosity pairs on compressed numerosity scales than on the linear scale, with the logarithmic scale showing the most uniform pattern among the tested scales. Per-animal analyses showed similar scale-dependent patterns in both pigeons (Supplementary Figure S4).

Across numerosity pairs, the QMD neuron population showed slightly higher discriminability than the QMI neuron population (Figure 5F). This tendency was also observed when the two pigeons were analyzed separately (Supplementary Figure S4). Consistent with this, sliding-window percent-explained variance (PEV; *ω*^2^) analysis revealed a higher peak *ω*^2^ value for the numerosity factor in the QMD population than in the QMI population (Figure 5G). In addition, the numerosity-coding window was markedly later in the QMD population (Figure 5G). The per-animal analyses also revealed similar PEV temporal dynamics in both pigeons (Supplementary Figure S4). The later coding window, together with the relatively higher discriminability of QMD neurons, may reflect top-down feedback modulation.

### 3.5. Time-Resolved Numerosity Discriminability and Population Decoding

**Figure 6:**
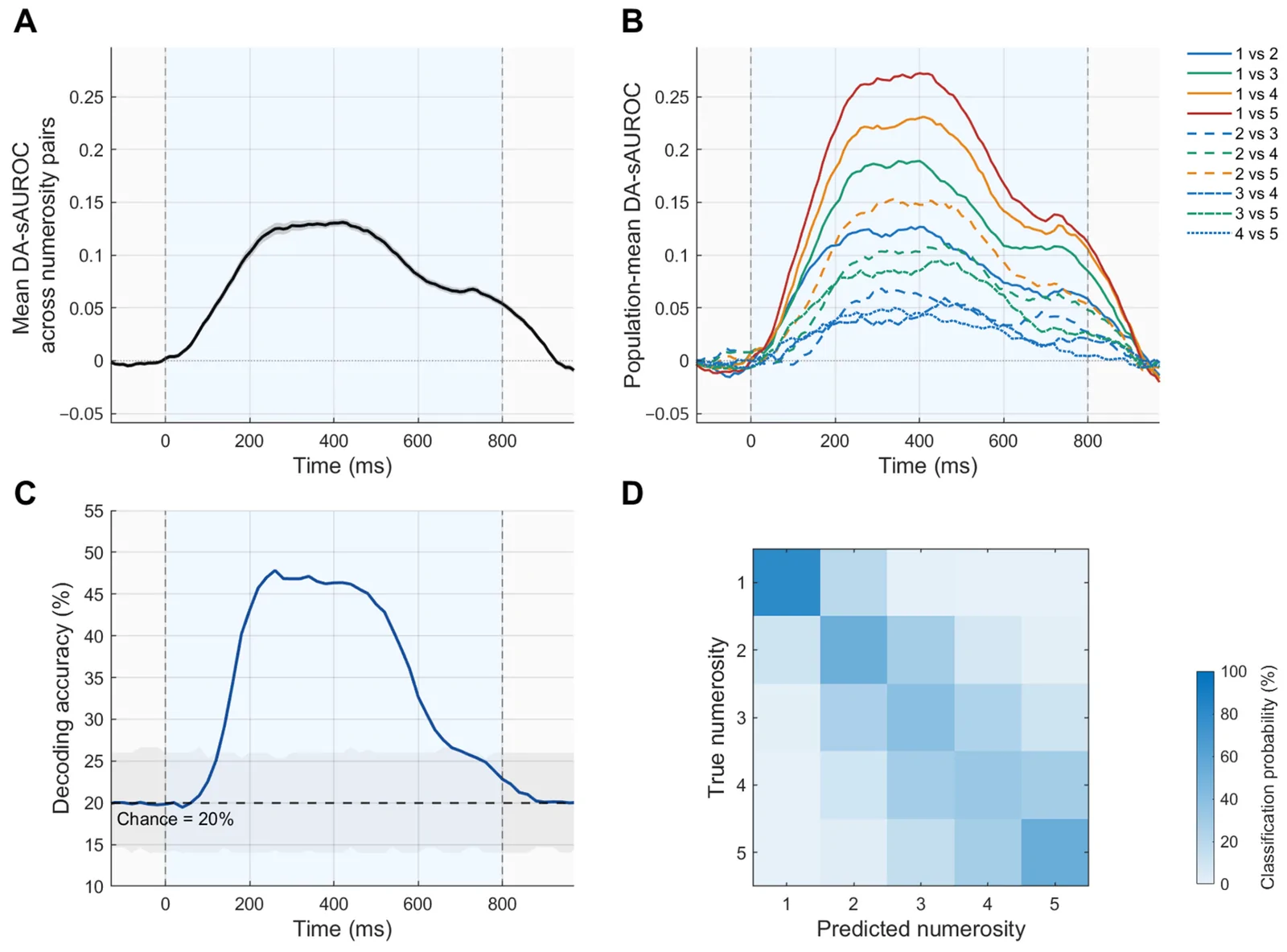
Time-resolved numerosity discriminability and population decoding. (**A**) Mean time-resolved DA-sAUROC across all 10 numerosity pairs in the combined QMI and QMD population (*n* = 191). Gray shading indicates SEM across neurons. (**B**) Population-mean time-resolved DA-sAUROC for each numerosity pair. (**C**) Mean time-resolved five-class SVM decoding accuracy across 100 resampling iterations. Blue shading represents SEM across resampling iterations. The gray shaded area indicates the 5th–95th percentile range of the shuffled-label decoding-accuracy distribution, and the horizontal dashed line indicates the theoretical chance level of 20%. (**D**) Row-normalized confusion matrix obtained from classifiers trained on mean neuronal responses within the predefined sample-related analysis interval, averaged across 1000 resampling iterations.

To examine the temporal evolution of numerosity discriminability across the combined QMI and QMD population, we calculated time-resolved DA-sAUROC for all 191 numerosity-selective neurons. Mean DA-sAUROC across the 10 numerosity pairs increased after sample onset, reached a broad maximum during the middle of the sample period, and then gradually declined (Figure 6A). Pair-specific time courses showed broadly similar temporal profiles, with discriminability generally increasing with numerical distance; the highest values were observed for the numerosity pair 1 versus 5 (Figure 6B).

Figure 6C shows the time course of population-level numerosity decoding during the sample period. Decoding accuracy began to increase rapidly at approximately 100 ms after sample onset and declined from approximately 500 ms onward. A continuous interval from 140 to 670 ms remained significant after correction for multiple comparisons across time (one-sided maximum-cluster-length permutation test, *p* = 0.001). Using mean neuronal responses within the predefined sample-related analysis period (50–900 ms), the classifier achieved an overall decoding accuracy of 51.6 ± 0.2% (mean ± SEM) across 1000 resampling iterations (Figure 6D). Classification probabilities in the confusion matrix were concentrated along the main diagonal, with errors occurring predominantly between neighboring numerosities.

## 4. Discussion

In this study, to investigate upstream neuronal processing of numerosity within the avian visual hierarchy, we recorded single-unit activity in the pigeon ENTO. We identified a subset of neurons that, within their respective selectivity time windows, encoded numerosity independently of the non-numerical feature controlled in the corresponding recording session and exhibited quasi-monotonic increasing (QMI) or quasi-monotonic decreasing (QMD) response profiles. Compared with QMI neurons, QMD neurons exhibited a markedly later numerosity-coding window and stronger category discriminability. At the population level, both neuronal populations showed evidence of compressed numerosity scaling.

Unlike the single-unit recordings used here, fMRI measures hemodynamic signals on a more macroscopic scale [49,50]. Using 7T fMRI, Paul et al. showed that responses in human early visual cortex (V1–V3) tracked first-harmonic aggregate Fourier power more closely than numerosity, and proposed that these responses provide feedforward signals from which more abstract numerosity representations can be derived [47]. Distinct from the retinotopic architecture of mammalian V1–V3, ENTO neurons are characterized by large receptive fields and little to no orderly retinotopy [29,30,51]. Following Paul et al.’s approach, we computed first-harmonic aggregate Fourier power for the numerosity stimuli (see Methods and Supplementary Figure S2). Statistical comparisons revealed that ENTO neuronal responses tracked numerosity more closely than first-harmonic aggregate Fourier power (Wilcoxon signed-rank test, *p* < 0.001, *n* = 191). While ENTO neurons encode numerosity information, it remains unclear whether this encoding capacity reflects an intrinsic functional property of the ENTO or is progressively shaped and stabilized by prolonged reinforcement-based training. The delayed match-to-numerosity task allowed us to record ENTO neuronal activity while pigeons actively extracted task-relevant numerosity information from visual stimuli. By contrast, paradigms in which numerosity is task-irrelevant are used to examine spontaneous numerosity coding. Task training can modulate neuronal response profiles by strengthening selectivity for task-relevant features while attenuating responses to task-irrelevant stimulus dimensions [52,53,54]. Consistent with this possibility, previous studies have reported spontaneous numerosity coding in approximately 12% of NCL neurons in crows performing a color-discrimination task and in approximately 11% of NCL neurons in young domestic chicks during passive stimulus presentation [18,20]. By comparison, studies in trained crows performing numerosity-related tasks have reported that numerosity-coding neurons account for approximately 18–24% of neurons in the NCL [16,17,37,55]. These proportions should be compared cautiously, because they may vary with the experimental paradigm, task phase, stimulus format, and selection criteria. Future studies using paradigms in which numerosity is task-irrelevant will be needed to determine whether spontaneous numerosity coding occurs in the pigeon ENTO and, if so, what proportion of neurons show such coding.

The relatively high proportion of numerosity-selective units observed in the ENTO in the present study (41%) may reflect functional differences between the ENTO and NCL. As a major visual processing region, the ENTO may contain a broader population of neurons modulated by task-relevant visual stimulus dimensions. By contrast, the NCL is a higher-order associative region involved in multiple cognitive functions, including working memory [56], rule-guided behavior [57], decision-making [58], and reward-related processing [59]. Numerosity-selective neurons may therefore constitute a relatively small subset of the NCL neuronal population. Differences in analytical approach may also have contributed to the relatively high proportion observed here: numerosity-selective units were identified using a sliding-window ANOVA in the present study, whereas some previous studies used fixed-epoch analyses.

At the level of static response profiles, QMD and QMI neurons resemble boundary-preferring neurons tuned to numerosities 1 and 5, respectively, within a labeled-line coding framework. However, the QMI and QMD populations differed markedly in their numerosity-coding windows and also showed differences in category discriminability, indicating a functional differentiation that is not fully captured by the conventional characterization of labeled-line responses based solely on preferred numerosity and distance-dependent attenuation. Moreover, some neurons in both populations exhibited coarse category-selective response profiles, with weak or absent discrimination among certain neighboring numerosities but pronounced response changes across specific boundaries separating adjacent numerosities. These step-like profiles are likewise not readily captured by conventional preferred-numerosity tuning and may reflect a coarser partitioning of numerical space. The coexistence of increasing and decreasing response patterns in the ENTO is broadly consistent with summation-coding accounts of numerosity processing [60,61]. Both increasing and decreasing monotonic responses have been reported in macaque LIP and interpreted as accumulator-like signals that may provide input to neurons encoding specific cardinal values [61]; computational models have likewise generated positive and negative summation-coding units [62]. Taken together, these findings suggest that numerosity-related response properties in the ENTO may provide a representational basis for the emergence of labeled-line coding in downstream associative regions.

Unlike the differential-outcomes delayed matching-to-sample task used by Johnston et al. [30], the present task did not assign different reward outcomes to different sample numerosities. Thus, QMI and QMD activity is unlikely to directly reflect sample-specific reward expectation. In addition, because match and non-match trials were pseudo-randomly interleaved and the choice display appeared only after the delay period, sample-related QMI and QMD activity is also unlikely to directly reflect a trial-type-specific decision process. QMI and QMD neurons showed sustained numerosity effects within their respective selectivity windows, with no detectable stimulus-format dependence in those windows. Nevertheless, the present analyses cannot determine whether this numerosity-related activity, particularly the delayed QMD activity, also contained task-related components such as reward expectation. Because such components were not tied to particular sample numerosities in the present task, they could shift the overall response level in a non-numerosity-specific manner, but they are unlikely by themselves to account for the systematic response differences among sample numerosities.

Previous lesion studies have shown that the entopallium plays an important role in a broad range of visual discrimination tasks, including discrimination of color [43], pattern [63], size [64], and natural or artificial visual categories [65]. The present study extends this line of work to visual numerosity discrimination by showing that single-neuron activity in the entopallium is modulated by the number of items in the stimuli. Importantly, unlike traditional visual categories, numerosity possesses an intrinsic metric structure, allowing differences between category levels to be quantitatively defined. The present study provides single-neuron evidence for how numerical category information is organized in the entopallium, thereby offering a quantitative entry point for understanding the coding principles underlying category-related processing in this region.

Population-level analyses showed that, in both the QMI and QMD neuronal populations, normalized response functions and category discriminability were better described by compressed numerosity scales than by a linear scale, with the logarithmic scale showing a modest overall advantage. This pattern is compatible with Fechner-like compression of numerosity in the ENTO. Furthermore, unlike the QMI population, the QMD population showed little numerosity-related variance during the early portion of the sample period. Later in the sample period, the variance explained by numerosity increased markedly and became the dominant component. This delayed onset of numerosity encoding in QMD neurons, coupled with their enhanced category discriminability, may reflect top-down feedback modulation. However, because the present study did not include lesion, inactivation, or pathway-specific interventions, this interpretation remains inferential. Future studies using such causal manipulations will be needed to directly test whether the delayed QMD activity depends on feedback-related mechanisms.

## 5. Conclusions

In summary, our findings show that the pigeon entopallium contains neurons that encode numerosity independently of the non-numerical feature controlled in the corresponding recording session and exhibit QMI or QMD response profiles. Notably, these two neuronal populations differed in their temporal dynamics and category discriminability: QMD neurons exhibited a later numerosity-coding window and stronger category discriminability than QMI neurons, a pattern that may reflect top-down feedback modulation. Further analyses showed that the response functions and category discriminability of both neuronal populations were better described by compressed rather than linear numerosity scales, with the logarithmic scale showing a modest overall advantage. Taken together, our findings help refine current theoretical frameworks for how numerosity information is extracted and represented along the avian visual processing hierarchy, offering a comparative basis for cross-species research. More broadly, this study advances our understanding of the neuronal mechanisms that support numerical competence.

## Figures and Tables

**Table 1:** Two-way ANOVA results for the example neurons shown in Figure 2 and Figure 3.

Figure Panel	Animal ID	Selectivity Window	Two-Way ANOVA Results		
			Numerosity	Stimulus Type	Interaction
Figure 2A	A	130–720 ms	*F*(4555) = 14.606, *p* < 0.001	*F*(1555) = 0.009, *p* = 0.926	*F*(4555) = 0.753, *p* = 0.556
Figure 2B	B	130–560 ms	*F*(4442) = 10.367, *p* < 0.001	*F*(1442) = 0.076, *p* = 0.783	*F*(4442) = 0.495, *p* = 0.74
Figure 2C	A	130–420 ms	*F*(4381) = 15.3, *p* < 0.001	*F*(1381) = 0.023, *p* = 0.881	*F*(4381) = 0.452, *p* = 0.771
Figure 2D	A	290–730 ms	*F*(4555) = 34.592, *p* < 0.001	*F*(1555) = 0.764, *p* = 0.382	*F*(4555) = 0.67, *p* = 0.613
Figure 2E	A	220–720 ms	*F*(4555) = 27.986, *p* < 0.001	*F*(1555) = 0.804, *p* = 0.37	*F*(4555) = 0.343, *p* = 0.849
Figure 2F	B	620–790 ms	*F*(4527) = 8.587, *p* < 0.001	*F*(1527) = 0.498, *p* = 0.48	*F*(4527) = 0.398, *p* = 0.81
Figure 3A	A	180–380 ms	*F*(4381) = 6.467, *p* < 0.001	*F*(1381) = 0.288, *p* = 0.592	*F*(4381) = 0.11, *p* = 0.979
Figure 3B	B	150–480 ms	*F*(4462) = 8.895, *p* < 0.001	*F*(1462) = 0.224, *p* = 0.637	*F*(4462) = 0.623, *p* = 0.646
Figure 3C	A	190–520 ms	*F*(4555) = 9.662, *p* < 0.001	*F*(1555) = 0.081, *p* = 0.777	*F*(4555) = 0.991, *p* = 0.412
Figure 3D	B	370–570 ms	*F*(4384) = 9.883, *p* < 0.001	*F*(1384) = 0.598, *p* = 0.44	*F*(4384) = 0.254, *p* = 0.907

## Data Availability

The data and analysis code supporting this study are available in the following GitHub repository: https://github.com/BrainSystemsLab/Data_and_Code_for_ENTO (accessed on 1 August 2026).

## Supplementary Materials

The following supporting information can be downloaded at: https://www.mdpi.com/article/10.3390/ani16162494/s1, Figure S1: Histological verification of electrode placement; Figure S2: Computation of first-harmonic aggregate Fourier power; Figure S3: Correct- and error-trial response profiles of QMI and QMD neurons; Figure S4: Per-animal analyses of numerosity discriminability and temporal dynamics in QMI and QMD neuron populations; Table S1: Per-animal summary of unit distribution and QMI/QMD response latencies; Table S2: Per-animal and pooled comparisons of scale-fitting metrics between the logarithmic scale and other candidate numerosity scales; Table S3: Per-animal and pooled comparisons of scale-fitting metrics between the linear scale and power-transformed compressed numerosity scales; Table S4: Two-way ANOVA results for QMI neurons within respective selectivity windows; Table S5: Two-way ANOVA results for QMD neurons within respective selectivity windows.
