# Supplementary material for: Categorical Representation of Numerosity in the Pigeon Entopallium: Coding Format and Temporal Dynamics
Source: Animals (Basel). 2026 Aug 11;16(16):2494. doi: 10.3390/ani16162494 (PMC13508866; doi:10.3390/ani16162494)
Supplement: Supplementary file 1 [file animals-16-02494-s001.zip › animals-4412213-supplementary.pdf]

Supplementary Materials

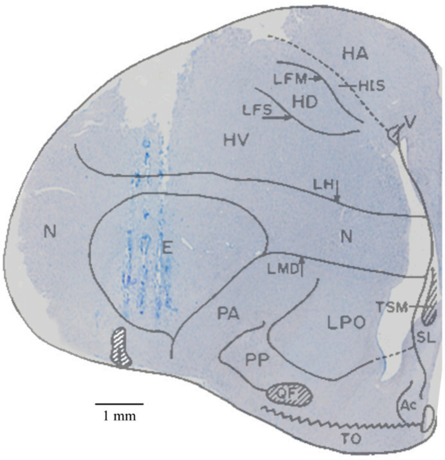

Figure S1. Histological verification of electrode placement. A representative coronal section (from Pigeon B) showing the electrode track within the entopallium (E), with anatomical boundaries delineated based on the Karten and Hodos atlas. Abbreviations: N, nidopallium; LPO, lobus parolfactorius; TO, tuberculum olfactorium; HA, hyperpallium apicale; HD, hyperpallium densocellulare; HV, hyperstriatum ventrale (mesopallium); PA, paleostriatum augmentatum; PP, paleostriatum primitivum.

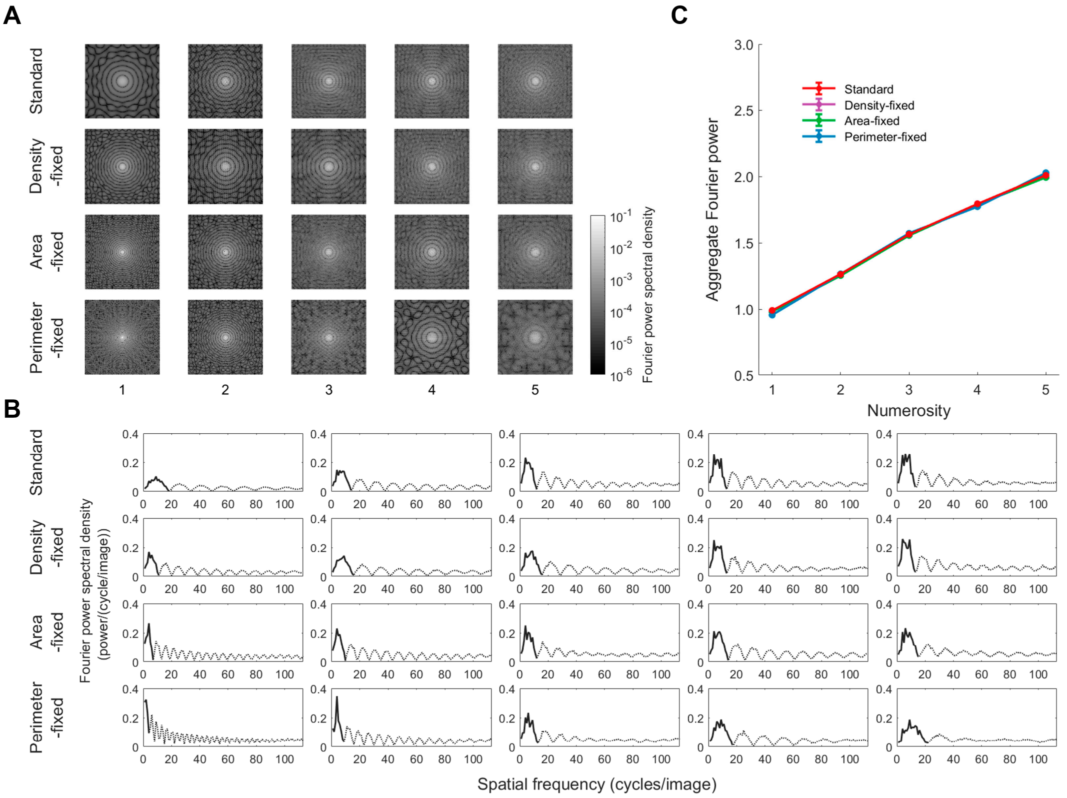

Figure S2. Computation of first-harmonic aggregate Fourier power. (**A**) Examples of two-dimensional Fourier transforms of the standard and three types of control stimuli. (**B**) Fourier power spectral density as a function of spatial frequency, collapsed over orientation, for the transformed images shown in (**A**). First-harmonic aggregate Fourier power for each image was computed by summing the power spectral density across spatial frequencies within the first harmonic (solid portion of each curve). (**C**) First-harmonic aggregate Fourier power for numerosities 1–5. Values are highly similar for the standard and the three control stimulus sets. Each point represents the mean first-harmonic aggregate Fourier power across 200 example stimuli. Error bars represent SEM.

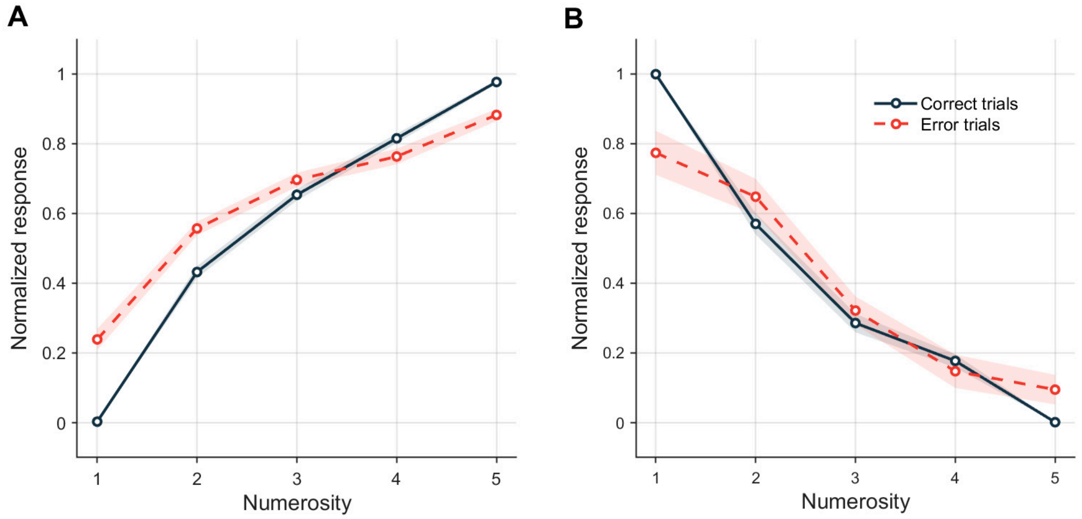

Figure S3. Correct- and error-trial response profiles of QMI and QMD neurons. Normalized response profiles of QMI (**A**) and QMD (**B**) neurons during correct trials (solid lines) and error trials (dashed lines). Lines represent population means, and shaded areas represent SEM. The mean magnitude of error-related modulation was 0.231 ± 0.095 for QMI neurons and 0.252 ± 0.081 for QMD neurons (mean ± SD), and the between-population difference was not statistically significant (Mann–Whitney U test, $p = 0.103$).

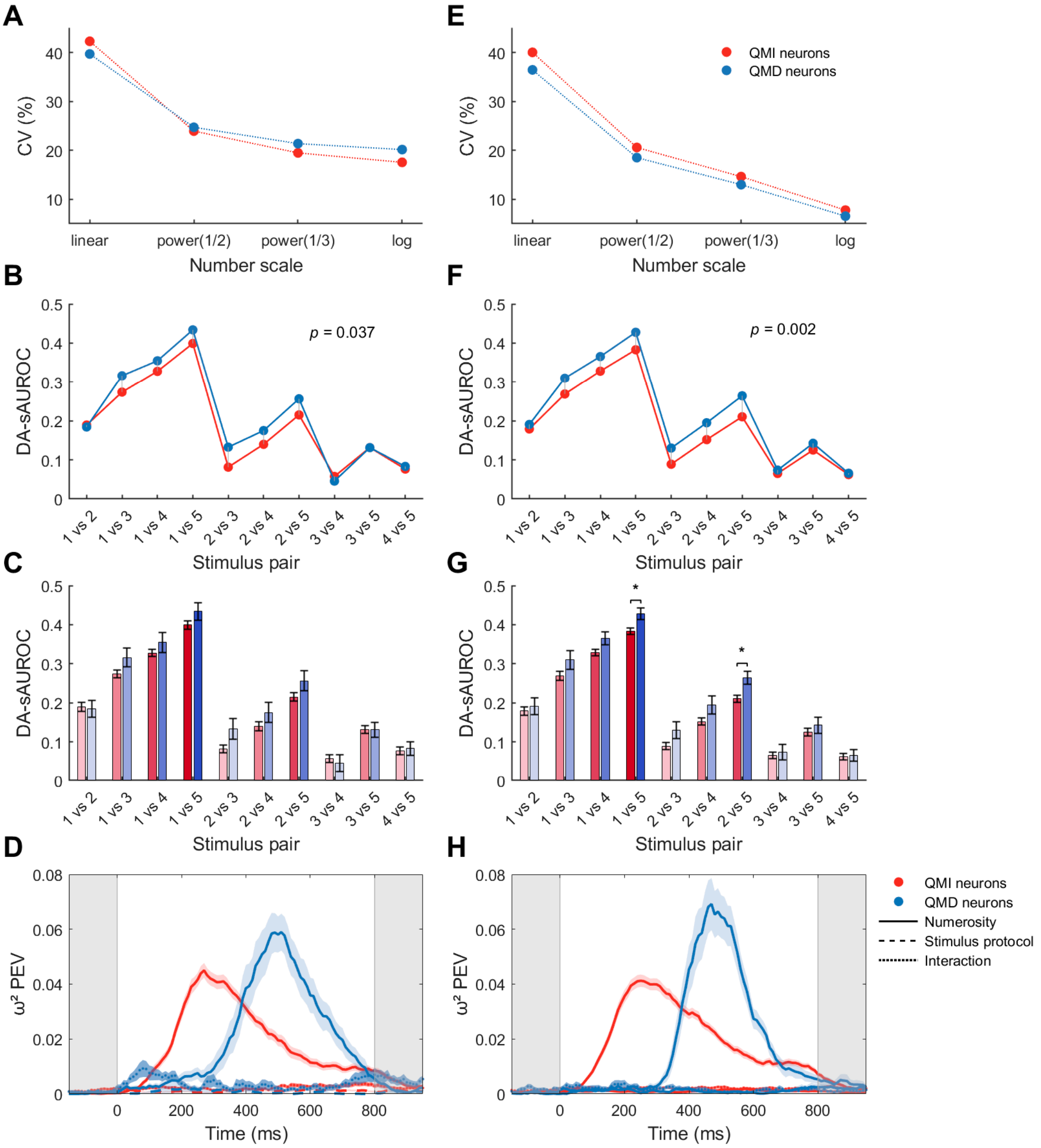

Figure S4. Per-animal analyses of numerosity discriminability and temporal dynamics in QMI and QMD neuron populations. (**A–D**) Results from Pigeon A. (**E–H**) Results from Pigeon B. (**A, E**) Coefficient of variation (CV) of per-unit-distance discriminability computed across all numerosity pairs for each number scale. Red and blue markers indicate QMI and QMD neuron populations, respectively. (**B, F**) Population-mean discriminability across the 10 numerosity pairs. Each point represents the mean DA-sAUROC for one numerosity pair. P values were obtained using paired Wilcoxon signed-rank tests across the 10 numerosity pairs. (**C, G**) Comparison of discriminability between QMI and QMD neuron populations for each numerosity pair. *$p < 0.05$, Mann–Whitney U test; error bars represent SEM. (**D, H**) Time courses of percent-explained variance (PEV; ω^2) for numerosity, stimulus protocol, and their interaction. Shaded areas around each curve represent SEM.

Table S1. Per-animal summary of unit distribution and QMI/QMD response latencies.

Animal ID	Recorded units	Numerosity-selective units	QMI	QMD	QMI latency	QMD latency	QMI vs. QMD latency
A	206	79 (38.4%)	62 (78.5%)	17 (21.5%)	196.8 ± 68.4 ms	379.4 ± 82.6 ms	$p < 0.001$
B	261	112 (42.9%)	93 (83.0%)	19 (17.0%)	190.9 ± 95.2 ms	400.5 ± 62.8 ms	$p < 0.001$

Note: Latencies are shown as mean ± SD. QMI and QMD latencies were compared using Mann–Whitney U tests.

Table S2. Per-animal and pooled comparisons of scale-fitting metrics between the logarithmic scale and other candidate numerosity scales.

Population and metric	Scale comparison	Pigeon A	Pigeon B	Pooled neurons
QMI R^2	linear vs. log	*** n = 62	*** n = 93	*** n = 155
	power(1/2) vs. log	** n = 62	* n = 93	*** n = 155
	power(1/3) vs. log	* n = 62	n.s. n = 93	* n = 155
QMI RMSE	linear vs. log	*** n = 62	*** n = 93	*** n = 155
	power(1/2) vs. log	** n = 62	* n = 93	*** n = 155
	power(1/3) vs. log	* n = 62	n.s. n = 93	** n = 155
QMD R^2	linear vs. log	** n = 17	** n = 19	*** n = 36
	power(1/2) vs. log	n.s. n = 17	n.s. n = 19	** n = 36
	power(1/3) vs. log	n.s. n = 17	n.s. n = 19	* n = 36
QMD RMSE	linear vs. log	** n = 17	* n = 19	*** n = 36
	power(1/2) vs. log	* n = 17	n.s. n = 19	** n = 36
	power(1/3) vs. log	n.s. n = 17	n.s. n = 19	* n = 36

Note: Paired Wilcoxon signed-rank tests were used to compare the logarithmic scale with each of the other candidate numerosity scales. n indicates the number of neurons included in each comparison. n.s., not significant; *$p < 0.05$; **$p < 0.01$; ***$p < 0.001$.

Table S3. Per-animal and pooled comparisons of scale-fitting metrics between the linear scale and power-transformed compressed numerosity scales.

Population and metric	Scale comparison	Pigeon A	Pigeon B	Pooled neurons
QMI R^2	linear vs. power (1/2)	*** n = 62	*** n = 93	*** n = 155
	linear vs. power (1/3)	*** n = 62	*** n = 93	*** n = 155
QMI RMSE	linear vs. power (1/2)	*** n = 62	*** n = 93	*** n = 155
	linear vs. power (1/3)	*** n = 62	*** n = 93	*** n = 155
QMD R^2	linear vs. power (1/2)	** n = 17	** n = 19	*** n = 36
	linear vs. power (1/3)	** n = 17	** n = 19	*** n = 36
QMD RMSE	linear vs. power (1/2)	** n = 17	** n = 19	*** n = 36
	linear vs. power (1/3)	** n = 17	** n = 19	*** n = 36

Note: Paired Wilcoxon signed-rank tests were used to compare the linear scale with each power-transformed scale. n indicates the number of neurons included in each comparison. $^{**}p < 0.01$; $^{***}p < 0.001$.

Table S4. Two-way ANOVA results for QMI neurons within respective selectivity windows

Unit	Animal ID	Selectivity window	Two-way ANOVA results		
			Numerosity	Stimulus type	Interaction
1	B	60–520 ms	F(4,506) = 16.313, p < 0.001	F(1,506) = 0.221, p = 0.639	F(4,506) = 0.444, p = 0.777
2	B	70–340 ms	F(4,473) = 10.084, p < 0.001	F(1,473) = 0.008, p = 0.93	F(4,473) = 0.928, p = 0.448
3	B	70–300 ms	F(4,473) = 8.995, p < 0.001	F(1,473) = 1.484, p = 0.224	F(4,473) = 0.346, p = 0.847
4	B	80–450 ms	F(4,493) = 14.429, p < 0.001	F(1,493) = 2.067, p = 0.151	F(4,493) = 1.237, p = 0.294
5	B	80–290 ms	F(4,307) = 9.777, p < 0.001	F(1,307) = 0.228, p = 0.633	F(4,307) = 0.765, p = 0.549
6	A	80–290 ms	F(4,449) = 7.78, p < 0.001	F(1,449) = 0.934, p = 0.334	F(4,449) = 0.583, p = 0.675
7	A	80–280 ms	F(4,412) = 5.233, p < 0.001	F(1,412) = 0.203, p = 0.653	F(4,412) = 0.798, p = 0.527
8	B	90–330 ms	F(4,432) = 9.139, p < 0.001	F(1,432) = 0.587, p = 0.444	F(4,432) = 0.928, p = 0.447
9	B	90–460 ms	F(4,506) = 7.992, p < 0.001	F(1,506) = 0.055, p = 0.815	F(4,506) = 0.154, p = 0.961
10	A	90–380 ms	F(4,363) = 10.358, p < 0.001	F(1,363) = 0.069, p = 0.793	F(4,363) = 0.744, p = 0.563
11	B	110–720 ms	F(4,554) = 17.986, p < 0.001	F(1,554) = 0.029, p = 0.865	F(4,554) = 0.211, p = 0.932
12	A	110–420 ms	F(4,497) = 8.04, p < 0.001	F(1,497) = 0.013, p = 0.91	F(4,497) = 0.23, p = 0.921
13	B	110–450 ms	F(4,432) = 14.829, p < 0.001	F(1,432) = 0.104, p = 0.747	F(4,432) = 1.481, p = 0.207
14	B	110–400 ms	F(4,432) = 7.387, p < 0.001	F(1,432) = 0.065, p = 0.799	F(4,432) = 1.048, p = 0.382
15	B	110–300 ms	F(4,457) = 6.551, p < 0.001	F(1,457) = 0.069, p = 0.793	F(4,457) = 0.507, p = 0.73
16	B	110–360 ms	F(4,506) = 8.987, p < 0.001	F(1,506) = 0.482, p = 0.488	F(4,506) = 0.607, p = 0.658
17	B	110–480 ms	F(4,307) = 12.755, p < 0.001	F(1,307) = 0.129, p = 0.72	F(4,307) = 0.925, p = 0.449
18	A	110–280 ms	F(4,363) = 5.295, p < 0.001	F(1,363) = 1.651, p = 0.2	F(4,363) = 0.256, p = 0.906
19	B	120–230 ms	F(4,450) = 5.497, p < 0.001	F(1,450) = 0.876, p = 0.35	F(4,450) = 0.72, p = 0.579
20	A	120–390 ms	F(4,476) = 7.75, p < 0.001	F(1,476) = 0.073, p = 0.787	F(4,476) = 1.254, p = 0.287
21	B	120–330 ms	F(4,486) = 8.961, p < 0.001	F(1,486) = 0.047, p = 0.828	F(4,486) = 1.251, p = 0.288
22	B	120–350 ms	F(4,486) = 10.015, p < 0.001	F(1,486) = 1.153, p = 0.283	F(4,486) = 0.939, p = 0.441
23	B	120–480 ms	F(4,442) = 10.718, p < 0.001	F(1,442) = 1.219, p = 0.27	F(4,442) = 0.379, p = 0.824
24	B	120–340 ms	F(4,561) = 6.56, p < 0.001	F(1,561) = 1.963, p = 0.162	F(4,561) = 0.369, p = 0.831
25	B	120–560 ms	F(4,506) = 11.907, p < 0.001	F(1,506) = 0.442, p = 0.506	F(4,506) = 0.914, p = 0.456
26	B	120–450 ms	F(4,506) = 15.944, p < 0.001	F(1,506) = 2.206, p = 0.138	F(4,506) = 0.375, p = 0.826
27	B	120–310 ms	F(4,473) = 7.965, p < 0.001	F(1,473) = 0.269, p = 0.604	F(4,473) = 0.73, p = 0.572
28	A	130–720 ms	F(4,555) = 14.606, p < 0.001	F(1,555) = 0.009, p = 0.926	F(4,555) = 0.753, p = 0.556
29	B	130–560 ms	F(4,442) = 10.367, p < 0.001	F(1,442) = 0.076, p = 0.783	F(4,442) = 0.495, p = 0.74
30	A	130–420 ms	F(4,381) = 15.3, p < 0.001	F(1,381) = 0.023, p = 0.881	F(4,381) = 0.452, p = 0.771
31	B	130–480 ms	F(4,450) = 9.885, p < 0.001	F(1,450) = 2.155, p = 0.143	F(4,450) = 0.162, p = 0.958
32	B	130–540 ms	F(4,554) = 16.209, p < 0.001	F(1,554) = 0.093, p = 0.761	F(4,554) = 0.713, p = 0.583
33	B	130–450 ms	F(4,307) = 7.796, p < 0.001	F(1,307) = 0.803, p = 0.371	F(4,307) = 0.452, p = 0.771
34	B	140–490 ms	F(4,527) = 9.489, p < 0.001	F(1,527) = 2.42, p = 0.12	F(4,527) = 0.851, p = 0.493
35	B	140–320 ms	F(4,538) = 4.986, p < 0.001	F(1,538) = 0.318, p = 0.573	F(4,538) = 0.183, p = 0.947
36	B	140–490 ms	F(4,489) = 10.959, p < 0.001	F(1,489) = 1.021, p = 0.313	F(4,489) = 1.263, p = 0.284
37	B	140–250 ms	F(4,489) = 4.765, p < 0.001	F(1,489) = 0.366, p = 0.545	F(4,489) = 0.433, p = 0.785
38	B	140–390 ms	F(4,489) = 8.073, p < 0.001	F(1,489) = 0.597, p = 0.44	F(4,489) = 1.921, p = 0.106

39	B	140–380 ms	$F(4,473) = 6.959, p < 0.001$	$F(1,473) = 1.158, p = 0.283$	$F(4,473) = 1.213, p = 0.305$
40	A	140–270 ms	$F(4,363) = 5.5, p < 0.001$	$F(1,363) = 0.974, p = 0.324$	$F(4,363) = 1.36, p = 0.247$
41	A	140–380 ms	$F(4,412) = 9.82, p < 0.001$	$F(1,412) = 2.351, p = 0.126$	$F(4,412) = 2.283, p = 0.06$
42	B	150–480 ms	$F(4,462) = 8.895, p < 0.001$	$F(1,462) = 0.224, p = 0.637$	$F(4,462) = 0.623, p = 0.646$
43	A	150–550 ms	$F(4,351) = 10.097, p < 0.001$	$F(1,351) = 2.022, p = 0.156$	$F(4,351) = 1.335, p = 0.256$
44	B	150–380 ms	$F(4,482) = 8.556, p < 0.001$	$F(1,482) = 0.568, p = 0.451$	$F(4,482) = 0.361, p = 0.836$
45	B	150–390 ms	$F(4,482) = 8.137, p < 0.001$	$F(1,482) = 0.671, p = 0.413$	$F(4,482) = 1.061, p = 0.375$
46	A	150–470 ms	$F(4,524) = 10.619, p < 0.001$	$F(1,524) = 1.157, p = 0.283$	$F(4,524) = 0.156, p = 0.96$
47	B	150–410 ms	$F(4,554) = 9.553, p < 0.001$	$F(1,554) = 2.506, p = 0.114$	$F(4,554) = 1.89, p = 0.111$
48	B	150–510 ms	$F(4,442) = 11.741, p < 0.001$	$F(1,442) = 3.289, p = 0.07$	$F(4,442) = 0.627, p = 0.644$
49	A	150–380 ms	$F(4,454) = 6.686, p < 0.001$	$F(1,454) = 1.197, p = 0.275$	$F(4,454) = 0.626, p = 0.644$
50	B	150–360 ms	$F(4,561) = 7.183, p < 0.001$	$F(1,561) = 4.278, p = 0.039$	$F(4,561) = 1.692, p = 0.15$
51	B	150–420 ms	$F(4,506) = 8.541, p < 0.001$	$F(1,506) = 0.949, p = 0.331$	$F(4,506) = 0.423, p = 0.792$
52	B	150–570 ms	$F(4,473) = 10.348, p < 0.001$	$F(1,473) = 0.387, p = 0.534$	$F(4,473) = 0.731, p = 0.571$
53	A	150–430 ms	$F(4,363) = 10.528, p < 0.001$	$F(1,363) = 0.067, p = 0.796$	$F(4,363) = 1.289, p = 0.274$
54	B	160–300 ms	$F(4,406) = 5.969, p < 0.001$	$F(1,406) = 0.334, p = 0.564$	$F(4,406) = 1.225, p = 0.3$
55	A	160–620 ms	$F(4,548) = 18.327, p < 0.001$	$F(1,548) = 0.004, p = 0.947$	$F(4,548) = 1.933, p = 0.104$
56	B	160–510 ms	$F(4,493) = 12.224, p < 0.001$	$F(1,493) = 0.078, p = 0.781$	$F(4,493) = 1.266, p = 0.282$
57	A	160–420 ms	$F(4,399) = 13.673, p < 0.001$	$F(1,399) = 0.649, p = 0.421$	$F(4,399) = 1.838, p = 0.121$
58	B	160–300 ms	$F(4,473) = 7.521, p < 0.001$	$F(1,473) = 0.063, p = 0.802$	$F(4,473) = 0.753, p = 0.556$
59	A	160–380 ms	$F(4,449) = 6.771, p < 0.001$	$F(1,449) = 0.021, p = 0.886$	$F(4,449) = 1.847, p = 0.119$
60	A	170–610 ms	$F(4,377) = 9.735, p < 0.001$	$F(1,377) = 0.005, p = 0.946$	$F(4,377) = 1.292, p = 0.273$
61	B	170–460 ms	$F(4,538) = 10.106, p < 0.001$	$F(1,538) = 1.097, p = 0.295$	$F(4,538) = 1.011, p = 0.401$
62	A	170–630 ms	$F(4,476) = 11.021, p < 0.001$	$F(1,476) = 0.572, p = 0.45$	$F(4,476) = 1.87, p = 0.115$
63	B	170–360 ms	$F(4,432) = 5.196, p < 0.001$	$F(1,432) = 0.057, p = 0.812$	$F(4,432) = 0.375, p = 0.826$
64	B	170–480 ms	$F(4,486) = 8.854, p < 0.001$	$F(1,486) = 0, p = 0.985$	$F(4,486) = 0.891, p = 0.469$
65	B	170–470 ms	$F(4,493) = 10.869, p < 0.001$	$F(1,493) = 0.315, p = 0.575$	$F(4,493) = 2.536, p = 0.039$
66	A	170–440 ms	$F(4,454) = 10.594, p < 0.001$	$F(1,454) = 0.037, p = 0.848$	$F(4,454) = 0.463, p = 0.763$
67	A	170–450 ms	$F(4,454) = 10.823, p < 0.001$	$F(1,454) = 0.179, p = 0.672$	$F(4,454) = 2.216, p = 0.066$
68	B	170–610 ms	$F(4,489) = 9.217, p < 0.001$	$F(1,489) = 0.056, p = 0.813$	$F(4,489) = 0.856, p = 0.49$
69	B	170–280 ms	$F(4,457) = 4.793, p < 0.001$	$F(1,457) = 0.591, p = 0.443$	$F(4,457) = 0.554, p = 0.696$
70	B	170–370 ms	$F(4,506) = 5.522, p < 0.001$	$F(1,506) = 0.048, p = 0.827$	$F(4,506) = 2.929, p = 0.021$
71	A	170–470 ms	$F(4,412) = 6.658, p < 0.001$	$F(1,412) = 0.763, p = 0.383$	$F(4,412) = 0.854, p = 0.491$
72	A	180–380 ms	$F(4,381) = 6.467, p < 0.001$	$F(1,381) = 0.288, p = 0.592$	$F(4,381) = 0.11, p = 0.979$
73	B	180–420 ms	$F(4,464) = 8.567, p < 0.001$	$F(1,464) = 0.131, p = 0.717$	$F(4,464) = 0.769, p = 0.546$
74	B	180–340 ms	$F(4,486) = 6.675, p < 0.001$	$F(1,486) = 1.073, p = 0.301$	$F(4,486) = 0.573, p = 0.682$
75	A	180–510 ms	$F(4,454) = 11.376, p < 0.001$	$F(1,454) = 0.072, p = 0.789$	$F(4,454) = 0.576, p = 0.68$
76	B	180–610 ms	$F(4,506) = 7.667, p < 0.001$	$F(1,506) = 4.867, p = 0.028$	$F(4,506) = 0.383, p = 0.821$
77	B	180–370 ms	$F(4,473) = 6.187, p < 0.001$	$F(1,473) = 0.069, p = 0.793$	$F(4,473) = 0.672, p = 0.612$
78	B	180–620 ms	$F(4,473) = 8.617, p < 0.001$	$F(1,473) = 0.004, p = 0.951$	$F(4,473) = 1.081, p = 0.365$
79	A	180–450 ms	$F(4,449) = 8.978, p < 0.001$	$F(1,449) = 0.821, p = 0.365$	$F(4,449) = 0.582, p = 0.676$

80	A	180–490 ms	$F(4,449) = 7.599, p < 0.001$	$F(1,449) = 0.394, p = 0.531$	$F(4,449) = 0.748, p = 0.56$
81	A	180–380 ms	$F(4,412) = 5.814, p < 0.001$	$F(1,412) = 0.011, p = 0.917$	$F(4,412) = 0.899, p = 0.464$
82	A	190–520 ms	$F(4,555) = 9.662, p < 0.001$	$F(1,555) = 0.081, p = 0.777$	$F(4,555) = 0.991, p = 0.412$
83	A	190–730 ms	$F(4,555) = 12.429, p < 0.001$	$F(1,555) = 4.955, p = 0.026$	$F(4,555) = 0.864, p = 0.485$
84	B	190–470 ms	$F(4,406) = 8.686, p < 0.001$	$F(1,406) = 0.232, p = 0.63$	$F(4,406) = 0.546, p = 0.702$
85	B	190–500 ms	$F(4,538) = 16.277, p < 0.001$	$F(1,538) = 1.582, p = 0.209$	$F(4,538) = 1.943, p = 0.102$
86	B	190–650 ms	$F(4,512) = 11.052, p < 0.001$	$F(1,512) = 0.551, p = 0.458$	$F(4,512) = 1.338, p = 0.255$
87	B	190–590 ms	$F(4,464) = 11.698, p < 0.001$	$F(1,464) = 0, p = 0.984$	$F(4,464) = 0.412, p = 0.8$
88	B	190–420 ms	$F(4,493) = 6.637, p < 0.001$	$F(1,493) = 0.041, p = 0.84$	$F(4,493) = 0.419, p = 0.795$
89	A	190–460 ms	$F(4,399) = 10.978, p < 0.001$	$F(1,399) = 0.65, p = 0.421$	$F(4,399) = 0.408, p = 0.803$
90	A	190–390 ms	$F(4,419) = 9.133, p < 0.001$	$F(1,419) = 0.453, p = 0.501$	$F(4,419) = 0.204, p = 0.936$
91	B	190–480 ms	$F(4,489) = 7.32, p < 0.001$	$F(1,489) = 0.05, p = 0.823$	$F(4,489) = 0.974, p = 0.421$
92	A	190–430 ms	$F(4,449) = 7.964, p < 0.001$	$F(1,449) = 0.111, p = 0.74$	$F(4,449) = 0.85, p = 0.494$
93	A	190–440 ms	$F(4,449) = 8.728, p < 0.001$	$F(1,449) = 0.032, p = 0.858$	$F(4,449) = 1.689, p = 0.151$
94	A	190–490 ms	$F(4,412) = 7.649, p < 0.001$	$F(1,412) = 0.694, p = 0.405$	$F(4,412) = 0.261, p = 0.903$
95	B	200–670 ms	$F(4,527) = 10.92, p < 0.001$	$F(1,527) = 0.707, p = 0.401$	$F(4,527) = 0.775, p = 0.542$
96	B	200–550 ms	$F(4,450) = 9.5, p < 0.001$	$F(1,450) = 0.109, p = 0.741$	$F(4,450) = 1.143, p = 0.336$
97	A	200–590 ms	$F(4,548) = 10.679, p < 0.001$	$F(1,548) = 3.839, p = 0.051$	$F(4,548) = 0.669, p = 0.614$
98	B	200–440 ms	$F(4,527) = 7.6, p < 0.001$	$F(1,527) = 2.82, p = 0.094$	$F(4,527) = 1.093, p = 0.359$
99	A	200–520 ms	$F(4,524) = 10.162, p < 0.001$	$F(1,524) = 1.596, p = 0.207$	$F(4,524) = 0.56, p = 0.692$
100	B	200–470 ms	$F(4,512) = 7.82, p < 0.001$	$F(1,512) = 0.28, p = 0.597$	$F(4,512) = 1.686, p = 0.152$
101	B	200–620 ms	$F(4,512) = 6.617, p < 0.001$	$F(1,512) = 0.744, p = 0.389$	$F(4,512) = 1.623, p = 0.167$
102	A	200–430 ms	$F(4,462) = 8.925, p < 0.001$	$F(1,462) = 0.042, p = 0.838$	$F(4,462) = 0.66, p = 0.62$
103	A	200–310 ms	$F(4,445) = 5.316, p < 0.001$	$F(1,445) = 0.019, p = 0.892$	$F(4,445) = 1.038, p = 0.387$
104	B	200–500 ms	$F(4,464) = 8.308, p < 0.001$	$F(1,464) = 0.593, p = 0.442$	$F(4,464) = 0.789, p = 0.532$
105	A	200–400 ms	$F(4,381) = 9.696, p < 0.001$	$F(1,381) = 0.02, p = 0.887$	$F(4,381) = 1.625, p = 0.167$
106	A	200–380 ms	$F(4,399) = 6.157, p < 0.001$	$F(1,399) = 1.097, p = 0.296$	$F(4,399) = 1.007, p = 0.403$
107	B	200–510 ms	$F(4,489) = 11.339, p < 0.001$	$F(1,489) = 0.006, p = 0.94$	$F(4,489) = 0.205, p = 0.936$
108	B	210–330 ms	$F(4,521) = 5.077, p < 0.001$	$F(1,521) = 0.314, p = 0.575$	$F(4,521) = 0.353, p = 0.842$
109	B	210–350 ms	$F(4,383) = 7.041, p < 0.001$	$F(1,383) = 0.332, p = 0.565$	$F(4,383) = 2.063, p = 0.085$
110	A	210–610 ms	$F(4,548) = 10.48, p < 0.001$	$F(1,548) = 2.378, p = 0.124$	$F(4,548) = 2.333, p = 0.055$
111	B	210–330 ms	$F(4,527) = 5.617, p < 0.001$	$F(1,527) = 2.094, p = 0.148$	$F(4,527) = 0.962, p = 0.428$
112	B	210–480 ms	$F(4,493) = 11.247, p < 0.001$	$F(1,493) = 0.522, p = 0.47$	$F(4,493) = 0.777, p = 0.541$
113	B	210–430 ms	$F(4,457) = 6.227, p < 0.001$	$F(1,457) = 0.575, p = 0.449$	$F(4,457) = 0.442, p = 0.779$
114	B	210–390 ms	$F(4,457) = 8.864, p < 0.001$	$F(1,457) = 2.417, p = 0.121$	$F(4,457) = 1.539, p = 0.19$
115	A	210–470 ms	$F(4,449) = 7.124, p < 0.001$	$F(1,449) = 0.049, p = 0.826$	$F(4,449) = 0.539, p = 0.707$
116	A	220–350 ms	$F(4,347) = 5.512, p < 0.001$	$F(1,347) = 0.031, p = 0.86$	$F(4,347) = 0.345, p = 0.847$
117	B	220–360 ms	$F(4,383) = 6.47, p < 0.001$	$F(1,383) = 0.054, p = 0.816$	$F(4,383) = 1.01, p = 0.402$
118	A	220–470 ms	$F(4,569) = 6.285, p < 0.001$	$F(1,569) = 0.079, p = 0.778$	$F(4,569) = 0.824, p = 0.51$
119	A	220–450 ms	$F(4,524) = 6.33, p < 0.001$	$F(1,524) = 0.105, p = 0.746$	$F(4,524) = 0.802, p = 0.524$
120	A	220–890 ms	$F(4,476) = 9.648, p < 0.001$	$F(1,476) = 0.027, p = 0.868$	$F(4,476) = 0.723, p = 0.576$

121	A	220–320 ms	$F(4,497) = 5.599, p < 0.001$	$F(1,497) = 0.789, p = 0.375$	$F(4,497) = 0.887, p = 0.471$
122	A	220–340 ms	$F(4,399) = 5.654, p < 0.001$	$F(1,399) = 1.259, p = 0.262$	$F(4,399) = 0.339, p = 0.851$
123	B	220–360 ms	$F(4,561) = 7.938, p < 0.001$	$F(1,561) = 0.47, p = 0.493$	$F(4,561) = 2.961, p = 0.019$
124	B	220–350 ms	$F(4,489) = 5.612, p < 0.001$	$F(1,489) = 0.001, p = 0.973$	$F(4,489) = 1.479, p = 0.207$
125	A	230–590 ms	$F(4,351) = 8.099, p < 0.001$	$F(1,351) = 0.01, p = 0.922$	$F(4,351) = 0.181, p = 0.948$
126	A	230–360 ms	$F(4,524) = 4.79, p < 0.001$	$F(1,524) = 0.414, p = 0.52$	$F(4,524) = 0.515, p = 0.725$
127	A	230–370 ms	$F(4,381) = 5.889, p < 0.001$	$F(1,381) = 0.403, p = 0.526$	$F(4,381) = 0.333, p = 0.856$
128	A	230–470 ms	$F(4,414) = 7.888, p < 0.001$	$F(1,414) = 1.349, p = 0.246$	$F(4,414) = 0.524, p = 0.718$
129	A	230–450 ms	$F(4,399) = 5.968, p < 0.001$	$F(1,399) = 0.032, p = 0.859$	$F(4,399) = 0.61, p = 0.655$
130	B	230–610 ms	$F(4,506) = 7.054, p < 0.001$	$F(1,506) = 0.009, p = 0.923$	$F(4,506) = 0.493, p = 0.741$
131	A	230–370 ms	$F(4,363) = 5.666, p < 0.001$	$F(1,363) = 0.782, p = 0.377$	$F(4,363) = 0.225, p = 0.924$
132	B	240–450 ms	$F(4,493) = 8.117, p < 0.001$	$F(1,493) = 0.551, p = 0.458$	$F(4,493) = 0.944, p = 0.438$
133	A	240–520 ms	$F(4,412) = 6.19, p < 0.001$	$F(1,412) = 1.273, p = 0.26$	$F(4,412) = 0.463, p = 0.763$
134	A	250–450 ms	$F(4,454) = 6.326, p < 0.001$	$F(1,454) = 1.48, p = 0.224$	$F(4,454) = 0.618, p = 0.65$
135	A	270–640 ms	$F(4,555) = 7.914, p < 0.001$	$F(1,555) = 1.197, p = 0.274$	$F(4,555) = 1.49, p = 0.204$
136	B	270–460 ms	$F(4,432) = 6.191, p < 0.001$	$F(1,432) = 1.214, p = 0.271$	$F(4,432) = 0.882, p = 0.474$
137	A	270–420 ms	$F(4,399) = 4.833, p < 0.001$	$F(1,399) = 0.28, p = 0.597$	$F(4,399) = 1.002, p = 0.406$
138	B	280–530 ms	$F(4,506) = 8.377, p < 0.001$	$F(1,506) = 0.195, p = 0.659$	$F(4,506) = 1.082, p = 0.365$
139	A	290–400 ms	$F(4,381) = 4.939, p < 0.001$	$F(1,381) = 0.01, p = 0.919$	$F(4,381) = 0.783, p = 0.537$
140	B	300–490 ms	$F(4,493) = 7.318, p < 0.001$	$F(1,493) = 0.609, p = 0.435$	$F(4,493) = 0.37, p = 0.83$
141	B	300–500 ms	$F(4,561) = 7.08, p < 0.001$	$F(1,561) = 2.57, p = 0.11$	$F(4,561) = 1.872, p = 0.114$
142	B	320–460 ms	$F(4,538) = 5.398, p < 0.001$	$F(1,538) = 1.389, p = 0.239$	$F(4,538) = 0.774, p = 0.542$
143	B	340–480 ms	$F(4,416) = 5.794, p < 0.001$	$F(1,416) = 0.003, p = 0.959$	$F(4,416) = 0.244, p = 0.913$
144	A	370–650 ms	$F(4,386) = 10.174, p < 0.001$	$F(1,386) = 0.052, p = 0.819$	$F(4,386) = 0.405, p = 0.805$
145	B	370–620 ms	$F(4,482) = 8.553, p < 0.001$	$F(1,482) = 0.282, p = 0.596$	$F(4,482) = 1.007, p = 0.403$
146	B	390–500 ms	$F(4,489) = 5.11, p < 0.001$	$F(1,489) = 0.837, p = 0.361$	$F(4,489) = 2.173, p = 0.071$
147	B	400–510 ms	$F(4,555) = 4.855, p < 0.001$	$F(1,555) = 3.276, p = 0.071$	$F(4,555) = 0.659, p = 0.621$
148	B	420–520 ms	$F(4,555) = 5.358, p < 0.001$	$F(1,555) = 0.422, p = 0.516$	$F(4,555) = 0.492, p = 0.741$
149	B	420–640 ms	$F(4,406) = 8.134, p < 0.001$	$F(1,406) = 1.927, p = 0.166$	$F(4,406) = 1.129, p = 0.342$
150	B	430–620 ms	$F(4,457) = 5.565, p < 0.001$	$F(1,457) = 1.505, p = 0.221$	$F(4,457) = 0.645, p = 0.63$
151	A	450–620 ms	$F(4,462) = 5.179, p < 0.001$	$F(1,462) = 0.488, p = 0.485$	$F(4,462) = 0.948, p = 0.436$
152	A	450–570 ms	$F(4,363) = 5.407, p < 0.001$	$F(1,363) = 2.832, p = 0.093$	$F(4,363) = 0.569, p = 0.685$
153	B	470–630 ms	$F(4,416) = 5.468, p < 0.001$	$F(1,416) = 0.929, p = 0.336$	$F(4,416) = 0.639, p = 0.635$
154	B	470–610 ms	$F(4,489) = 4.604, p < 0.01$	$F(1,489) = 1.927, p = 0.166$	$F(4,489) = 0.77, p = 0.545$
155	B	510–760 ms	$F(4,555) = 11.62, p < 0.001$	$F(1,555) = 0.15, p = 0.698$	$F(4,555) = 0.325, p = 0.861$

Note: Neurons are sorted by the onset of their respective selectivity windows used for response analyses. The order of neurons corresponds one-to-one to the rows in the upper panels (QMI) of Figure 4A and 4B. The significance threshold for all ANOVA effects was predefined as $p < 0.01$.

Table S5. Two-way ANOVA results for QMD neurons within respective selectivity windows

Unit	Animal ID	Selectivity window	Two-way ANOVA results		
			Numerosity	Stimulus type	Interaction
1	A	220–720 ms	$F(4,555) = 27.986, p < 0.001$	$F(1,555) = 0.804, p = 0.37$	$F(4,555) = 0.343, p = 0.849$
2	A	230–520 ms	$F(4,555) = 10.022, p < 0.001$	$F(1,555) = 0.122, p = 0.727$	$F(4,555) = 0.338, p = 0.852$
3	A	290–730 ms	$F(4,555) = 34.592, p < 0.001$	$F(1,555) = 0.764, p = 0.382$	$F(4,555) = 0.67, p = 0.613$
4	A	320–610 ms	$F(4,555) = 15.099, p < 0.001$	$F(1,555) = 0.611, p = 0.435$	$F(4,555) = 0.241, p = 0.915$
5	A	340–530 ms	$F(4,324) = 6.875, p < 0.001$	$F(1,324) = 0.12, p = 0.73$	$F(4,324) = 1.243, p = 0.292$
6	A	340–600 ms	$F(4,569) = 7.449, p < 0.001$	$F(1,569) = 0.025, p = 0.874$	$F(4,569) = 1.168, p = 0.324$
7	B	350–650 ms	$F(4,482) = 15.438, p < 0.001$	$F(1,482) = 0.007, p = 0.931$	$F(4,482) = 0.431, p = 0.786$
8	B	350–610 ms	$F(4,368) = 19.132, p < 0.001$	$F(1,368) = 0.696, p = 0.405$	$F(4,368) = 1.321, p = 0.262$
9	A	360–560 ms	$F(4,396) = 9.461, p < 0.001$	$F(1,396) = 0.004, p = 0.95$	$F(4,396) = 1.219, p = 0.302$
10	B	360–670 ms	$F(4,462) = 13.963, p < 0.001$	$F(1,462) = 1.719, p = 0.19$	$F(4,462) = 0.276, p = 0.894$
11	A	360–720 ms	$F(4,402) = 16.313, p < 0.001$	$F(1,402) = 1.557, p = 0.213$	$F(4,402) = 1.318, p = 0.263$
12	B	360–670 ms	$F(4,521) = 17.603, p < 0.001$	$F(1,521) = 0.655, p = 0.419$	$F(4,521) = 0.646, p = 0.63$
13	B	360–590 ms	$F(4,383) = 7.716, p < 0.001$	$F(1,383) = 0.051, p = 0.822$	$F(4,383) = 0.232, p = 0.92$
14	B	370–570 ms	$F(4,384) = 9.883, p < 0.001$	$F(1,384) = 0.598, p = 0.44$	$F(4,384) = 0.254, p = 0.907$
15	B	370–570 ms	$F(4,495) = 9.07, p < 0.001$	$F(1,495) = 1.289, p = 0.257$	$F(4,495) = 0.714, p = 0.583$
16	B	370–540 ms	$F(4,368) = 9.897, p < 0.001$	$F(1,368) = 1.13, p = 0.288$	$F(4,368) = 0.592, p = 0.669$
17	B	380–720 ms	$F(4,482) = 11.93, p < 0.001$	$F(1,482) = 0.162, p = 0.688$	$F(4,482) = 1.359, p = 0.247$
18	B	380–660 ms	$F(4,521) = 11.379, p < 0.001$	$F(1,521) = 0.111, p = 0.739$	$F(4,521) = 1.039, p = 0.386$
19	A	390–580 ms	$F(4,555) = 7.221, p < 0.001$	$F(1,555) = 0.077, p = 0.781$	$F(4,555) = 1.751, p = 0.137$
20	B	390–650 ms	$F(4,507) = 9.4, p < 0.001$	$F(1,507) = 0.004, p = 0.952$	$F(4,507) = 0.237, p = 0.917$
21	B	390–510 ms	$F(4,351) = 4.601, p < 0.01$	$F(1,351) = 0.02, p = 0.889$	$F(4,351) = 1.07, p = 0.371$
22	B	400–590 ms	$F(4,450) = 7.808, p < 0.001$	$F(1,450) = 1.714, p = 0.191$	$F(4,450) = 0.184, p = 0.947$
23	B	400–620 ms	$F(4,368) = 15.961, p < 0.001$	$F(1,368) = 2.454, p = 0.118$	$F(4,368) = 1.448, p = 0.217$
24	A	410–520 ms	$F(4,347) = 5.088, p < 0.001$	$F(1,347) = 0.445, p = 0.505$	$F(4,347) = 0.838, p = 0.502$
25	B	410–640 ms	$F(4,406) = 5.465, p < 0.001$	$F(1,406) = 0.081, p = 0.776$	$F(4,406) = 0.853, p = 0.493$
26	A	420–670 ms	$F(4,386) = 7.949, p < 0.001$	$F(1,386) = 0.034, p = 0.853$	$F(4,386) = 0.505, p = 0.732$
27	B	420–610 ms	$F(4,507) = 10.395, p < 0.001$	$F(1,507) = 0.067, p = 0.796$	$F(4,507) = 0.41, p = 0.801$
28	A	430–590 ms	$F(4,351) = 6.03, p < 0.001$	$F(1,351) = 0.002, p = 0.964$	$F(4,351) = 0.256, p = 0.906$
29	A	440–560 ms	$F(4,386) = 4.829, p < 0.001$	$F(1,386) = 0.083, p = 0.774$	$F(4,386) = 0.511, p = 0.728$
30	A	460–570 ms	$F(4,351) = 5.535, p < 0.001$	$F(1,351) = 0.053, p = 0.818$	$F(4,351) = 0.584, p = 0.675$
31	B	460–660 ms	$F(4,351) = 11.029, p < 0.001$	$F(1,351) = 0.535, p = 0.465$	$F(4,351) = 0.716, p = 0.582$
32	A	470–590 ms	$F(4,351) = 4.845, p < 0.001$	$F(1,351) = 0.299, p = 0.585$	$F(4,351) = 0.602, p = 0.661$
33	B	470–660 ms	$F(4,507) = 8.956, p < 0.001$	$F(1,507) = 0.065, p = 0.799$	$F(4,507) = 0.252, p = 0.909$
34	A	480–710 ms	$F(4,347) = 7.49, p < 0.001$	$F(1,347) = 0.006, p = 0.939$	$F(4,347) = 1.554, p = 0.186$
35	A	490–840 ms	$F(4,351) = 9.527, p < 0.001$	$F(1,351) = 0.896, p = 0.345$	$F(4,351) = 0.508, p = 0.73$
36	B	620–790 ms	$F(4,527) = 8.587, p < 0.001$	$F(1,527) = 0.498, p = 0.48$	$F(4,527) = 0.398, p = 0.81$

Note: Neurons are sorted by the onset of their respective selectivity windows used for response analyses. The order of neurons corresponds one-to-one to the rows in the lower panels (QMD) of Figure 4A and 4B. The significance threshold for all ANOVA effects was predefined as $p < 0.01$.
